# Supplementary material for: Genetic circuit characterization by inferring RNA polymerase movement and ribosome usage
Source: Nat Commun. 2020 Oct 5;11:5001. doi: 10.1038/s41467-020-18630-2 (PMC7536230; doi:10.1038/s41467-020-18630-2)
Supplement: Supplementary file 1 — Supplementary Information [file 41467_2020_18630_MOESM1_ESM.pdf]

Supplementary material for:

Genetic circuit characterization by inferring RNA polymerase movement and ribosome usage

Espah Borujeni et al.

Table of Contents:

I. Supplementary Figures
Supplementary Figure 1: Plasmid maps.
Supplementary Figure 2: Size distribution of the mapped fragments.
Supplementary Figure 3: Comparison of conventional and end-enriching RNA-seq methods.
Supplementary Figure 4: tRNA transcription.
Supplementary Figure 5: Conversion of arbitrary units to RPU for RNA-seq data.
Supplementary Figure 6: Comparison of transcription and translation between native and circuit genes.
Supplementary Figure 7: Incorrect annotated TSS.
Supplementary Figure 8: Identification of σ70 motifs in cryptic promoter sequences.
Supplementary Figure 9: Evidence for a cryptic antisense promoter in the ribozymes.
Supplementary Figure 10: Importance of ribozyme in sensor and gate characterizations.
Supplementary Figure 11: Evidence of transcriptional attenuation.
Supplementary Figure 12: Evidence for translational errors.
Supplementary Figure 13: Calculation of ribozyme cleavage efficiency (CE).
Supplementary Figure 14: Comparison of ribozyme cleavage efficiency (CE) and ΔG of RNA folding.
Supplementary Figure 15: The translation efficiency (TE) of genes across circuit states.
Supplementary Figure 16: Identification of the transcriptional termination site (TTS).
Supplementary Figure 17: Putative off-target *hlyIIR* binding sites in the genome.
Supplementary Figure 18: Boolean simulation of gate failures.
Supplementary Figure 19: Fitting the gate response functions to omics data.
Supplementary Figure 20: Dynamic modelling of genetic circuit using parameters extracted from omics data.
Supplementary Figure 21: Estimated proteome fraction of the circuit and native DNA-binding proteins.
Supplementary Figure 22: Expression of major chaperons in *E. coli* as a function of circuit proteome fraction.
Supplementary Figure 23: An example of gated flow cytometry data.
Supplementary Figure 24: Sense and antisense transcription profiles.
Supplementary Figure 25: Ribosome occupancy per transcript on both sense and antisense strands.

II. Supplementary Tables
Supplementary Table 1: Sequences of cryptic sense and antisense promoters.
Supplementary Table 2: Genes upregulated by higher resource usage.
Supplementary Table 3: Genes downregulated by higher resource usage.
Supplementary Table 4: Plasmid sequences.
Supplementary Table 5: Genetic part sequences.

III. Supplementary References

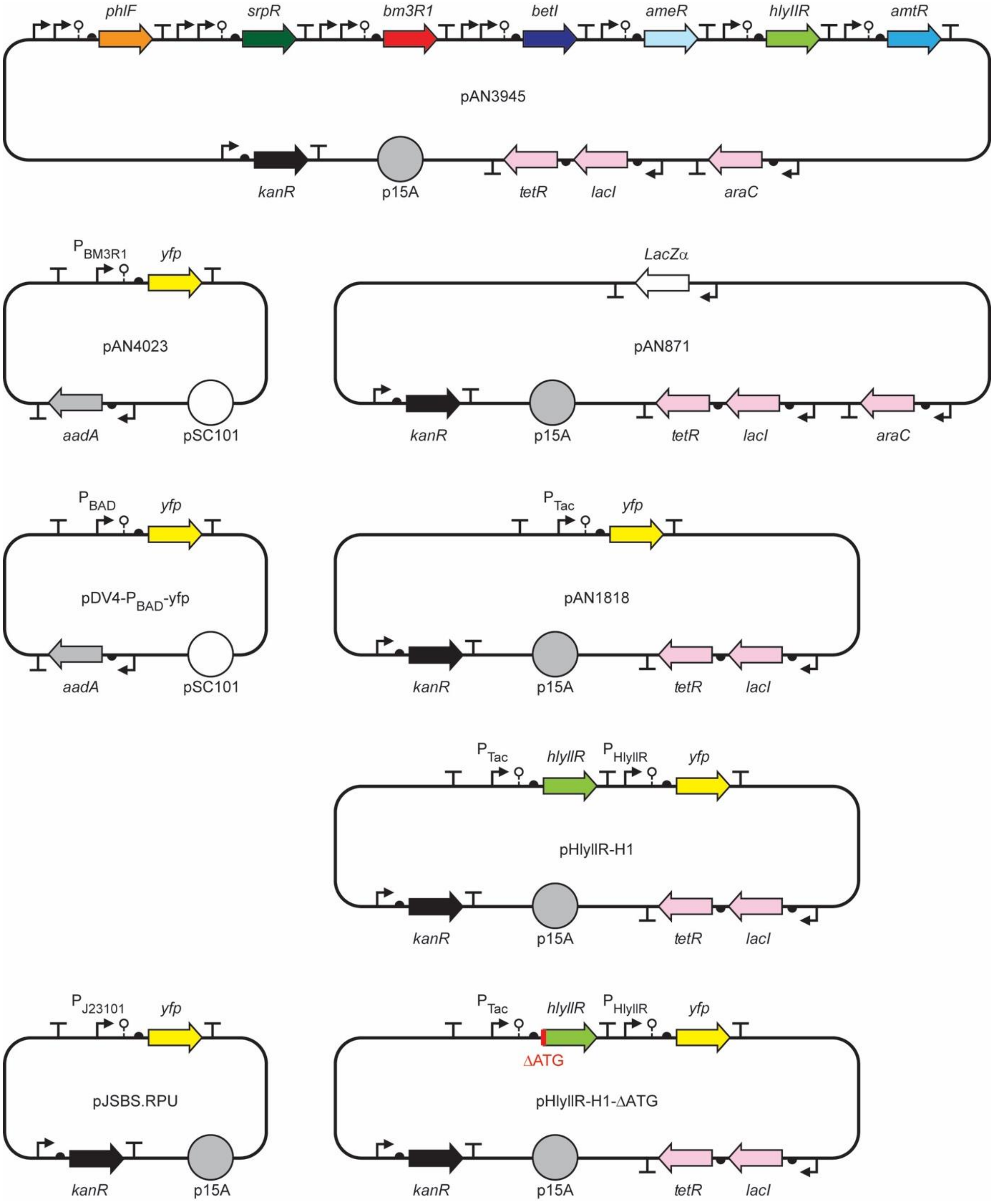

Supplementary Figure 1: **Plasmid maps.** The following plasmids were taken from previous works: pAN3945, pAN4023, pAN1818, and pHlyIIR-H1 from Nielsen *et al*[1]; pJSBS.RPU from Meyer *et al*[2]; and pAN871 from Andrews *et al*[3]. Plasmids pDV4-P_{BAD}-yfp and pHlyIIR-H1-ΔATG were built as part of this work. Plasmids and part sequences are provided in Supplementary Tables 4 and 5. Large circles are plasmid origins, and black and grey filled arrows are antibiotic resistant markers.

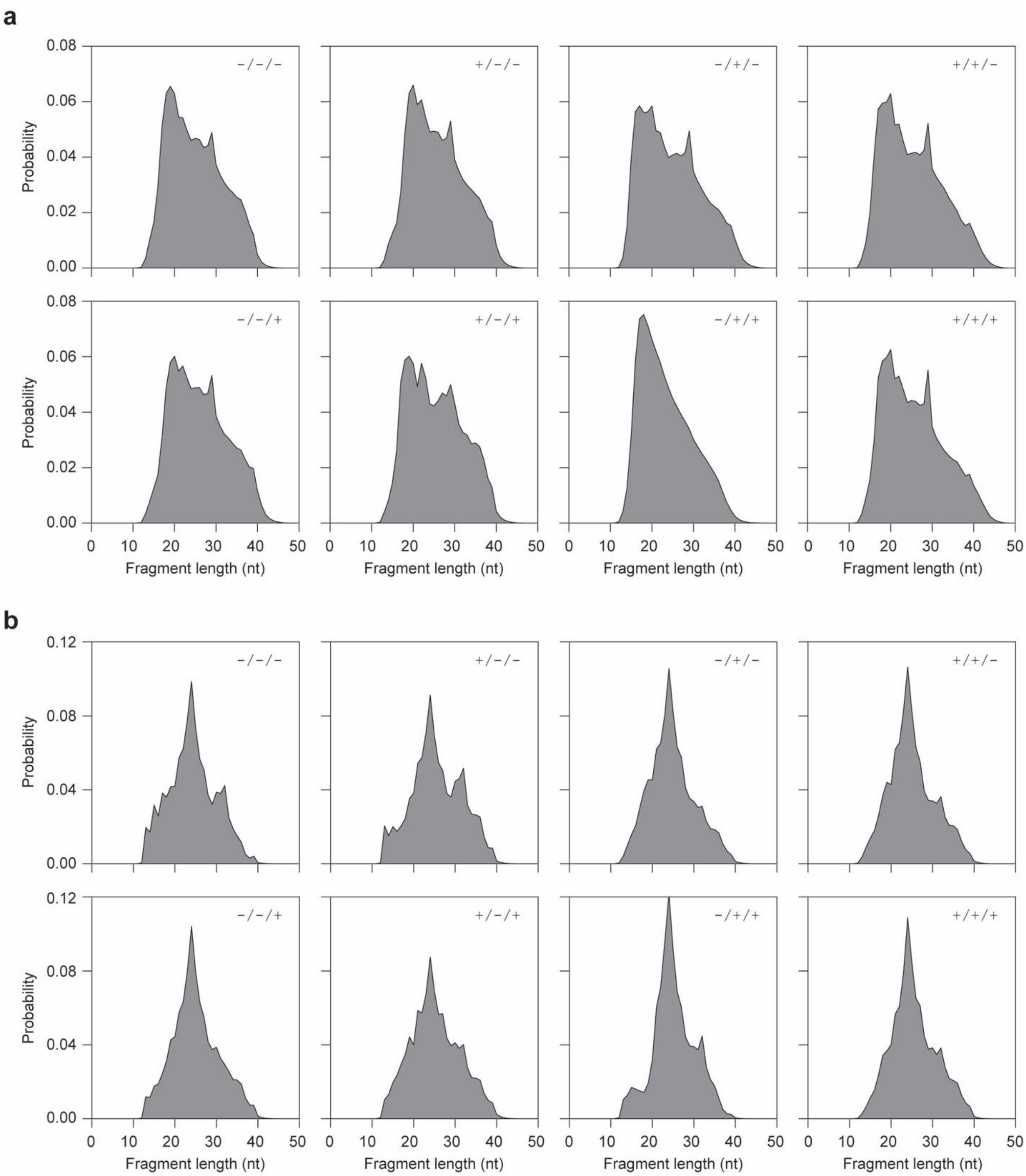

Supplementary Figure 2: **Size distribution of the mapped fragments.** Distribution of the mapped fragments from **(a)** RNA-seq and **(b)** ribosome profiling experiments across eight induction states are shown (Methods). For each sequencing experiment, all raw fragments that were mapped to the reference sequences (*E. coli* DH10B genome and plasmids) were collected and their length distribution was generated. The circuit state is indicated by the presence or absence of the IPTG/aTc/Ara inducers (top right of each graph). Source data are provided as a Source Data file.

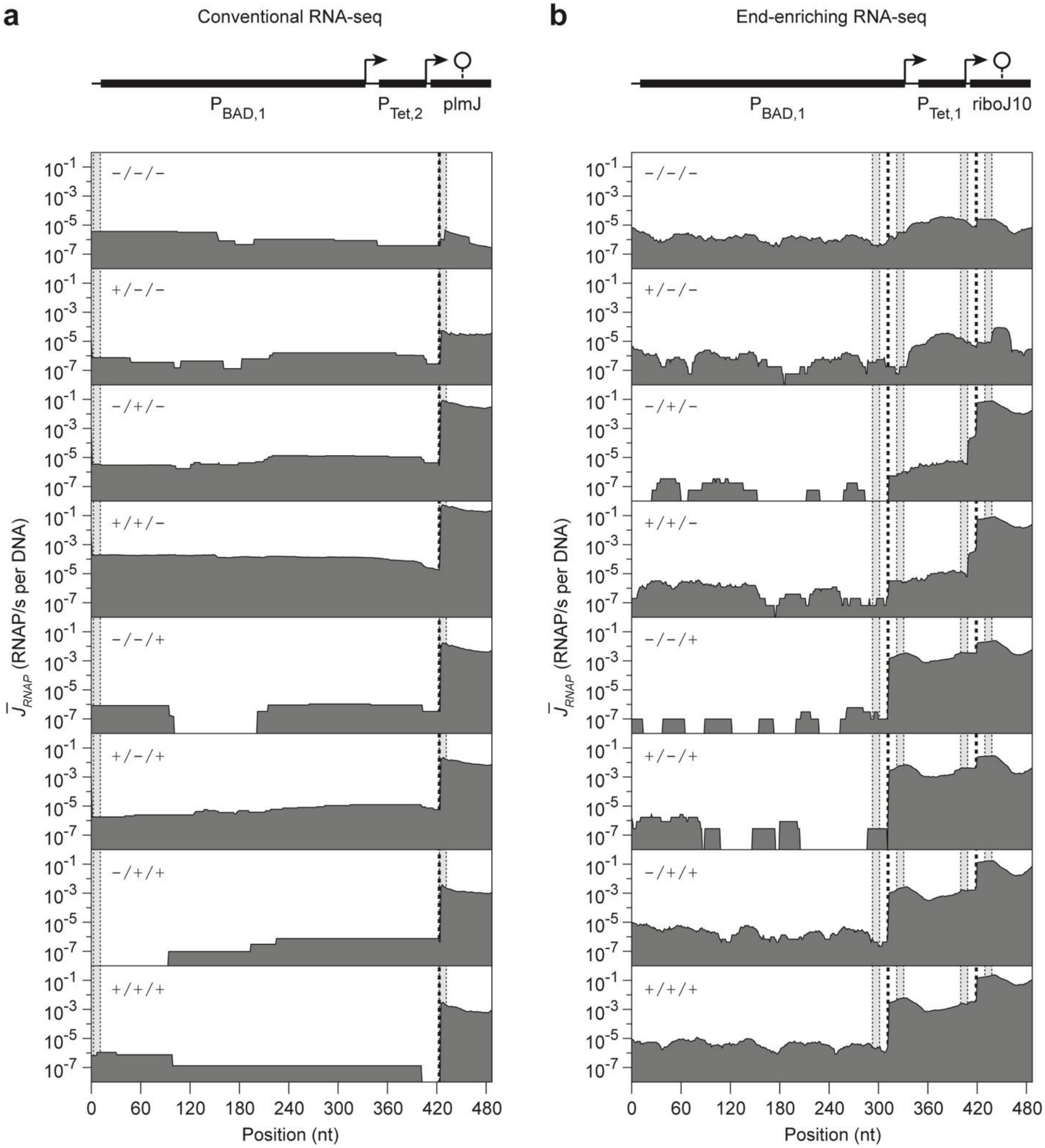

<u>Supplementary Figure 3:</u> **Comparison of conventional and end-enriching RNA-seq methods.** The transcripts are shown when the average fragment size is 280 nt **(a)** and 24 nt **(b)** (Methods). Data in **(a)** are taken from Gorochowski *et al*[4]. The TSSs are indicated by the thick dashed lines and the regions used to calculate the promoter strength are in grey. Note that effects due to the upstream promoter and downstream ribozyme are obscured when the fragment size is large. The circuit state is shown in the upper left of each profile: IPTG/aTc/Ara. Source data are provided as a Source Data file.

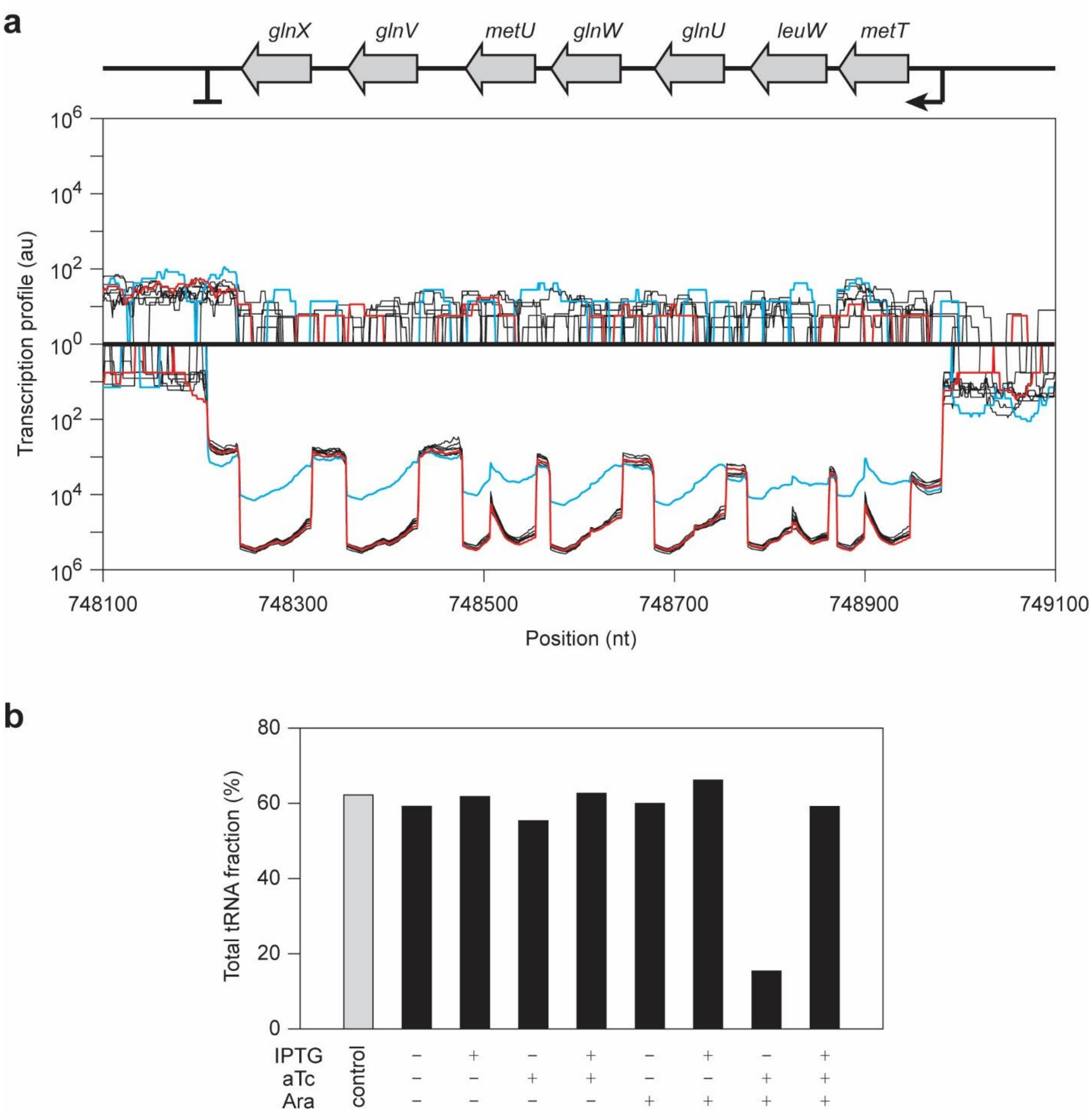

<u>Supplementary Figure 4:</u> **tRNA transcription. (a)** Transcription profiles around a tRNA operon are shown on both sense (top) and antisense (bottom) strands of the *E. coli* DH10B genome. The black lines correspond to data from the eight circuit states. The light blue line shows state -/+/+ (IPTG/aTc/Ara). The red line is *E. coli* cell with empty plasmid backbones (pAN871 and pDV4-P_{BAD}-yfp). Transcription profiles are in arbitrary units, and have been normalized by the total mapped nucleotides that include tRNAs (Methods). **(b)** The fraction of total tRNA in transcriptome is shown for the eight circuit states and the control (*E. coli* cell with empty plasmid backbones (pAN871 and pDV4-P_{BAD}-yfp)). Fractions are calculated as the ratio of total nucleotides mapped to the tRNAs over the total mapped nucleotides in the sample. Each bar is the value from one biological replicate. The state with lowest tRNA fraction (-/+/+) corresponds with the light blue line in **(a)**. Source data are provided as a Source Data file.

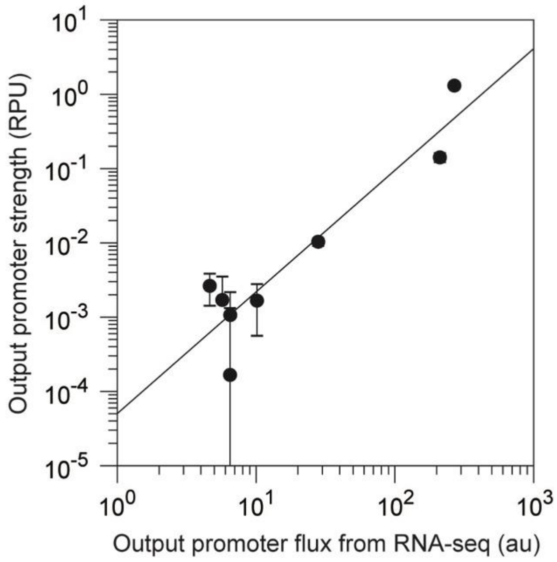

Supplementary Figure 5: **Conversion of arbitrary units to RPU for RNA-seq data.** To do this conversion, we made use of the fact that we know the output of the circuit (P_{BM3R1}) in RPU for the eight combinations of inducers (Figure 1c) (Methods). We also have the strength of this promoter extracted from the RNA-seq data. These data are plotted above and fit to a power law equation: $y = (5.05 \times 10^{-5}) x^{1.64}$ ($R^2 = 0.87$, p-value = 0.0008). The RPU values in y-axis are presented as the averages +/- standard deviations calculated from three replicates performed on different days. Source data are provided as a Source Data file.

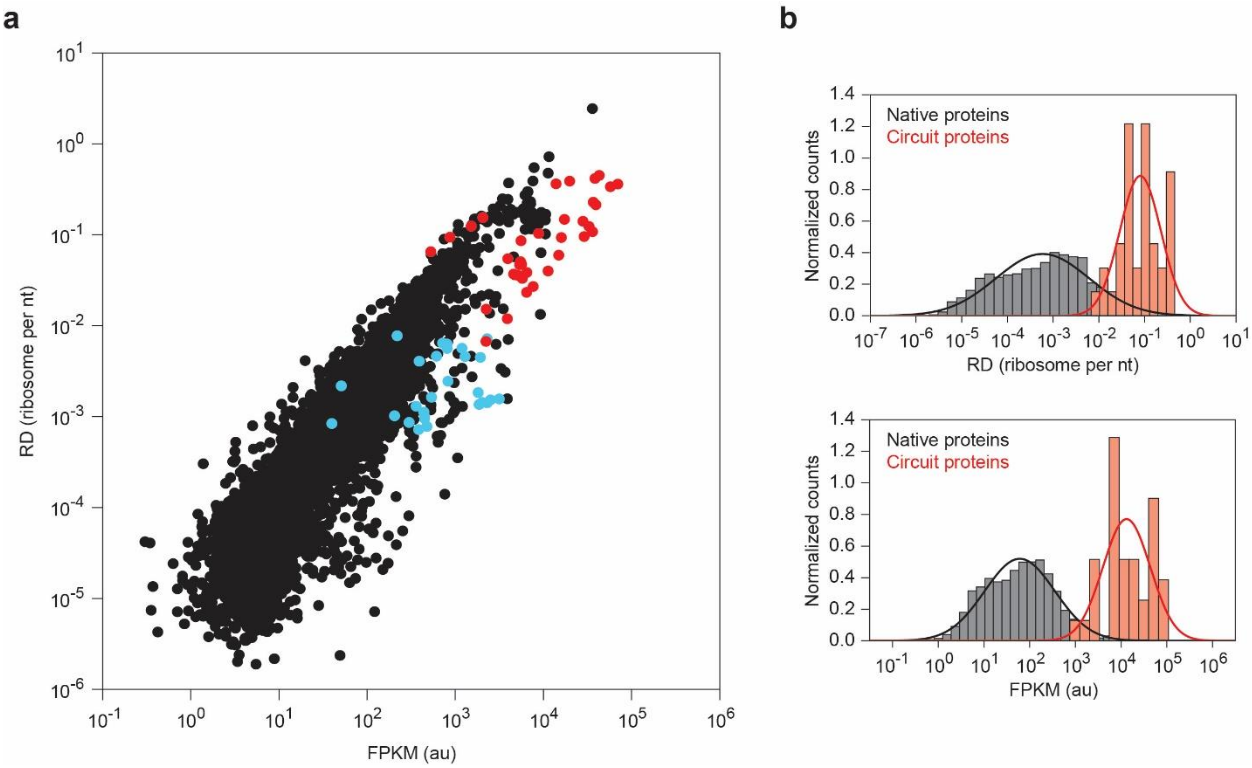

<u>Supplementary Figure 6:</u> Comparison of transcription and translation between native and circuit genes. (a) Each black dot is a native genome-encoded gene, measured using the control cell (Methods). The RD and FPKM of the circuit repressor genes across all circuit states are shown in blue if the gate is on (promoter controlling the repressor gene is off) and red if it is off (promoter controlling the repressor gene is on). **(b)** Distribution of RD and FPKM of native genes shown in **a** are compared with those of circuit repressor genes when their corresponding gate is off (repressor gene is transcribed). Histogram bars show the normalized counts (distribution) of each RD and FPKM within the data shown in **a**, and lines are the normal distribution fit to the histograms calculated using stats.norm function of scipy in Python. Source data are provided as a Source Data file.

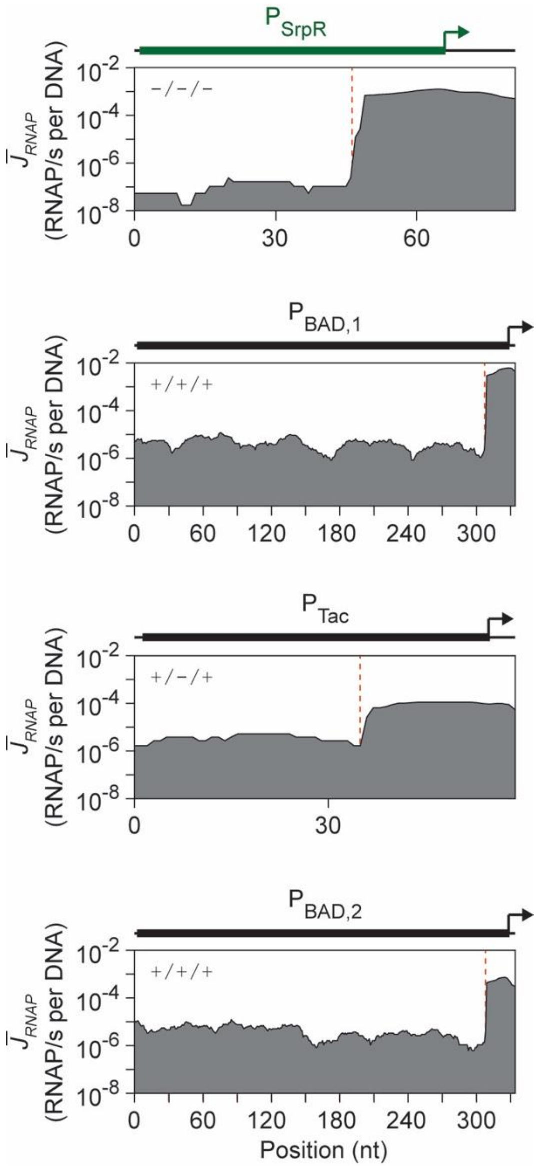

<u>Supplementary Figure 7:</u> **Incorrect annotated TSS.** The annotated positions are shown as the bent arrows, whereas the experimentally-determined positions are shown as red dashed lines. The profiles shown for P_{SrpR}, $P_{BAD,1}$, P_{Tac}, and $P_{BAD,2}$ are from states -/-/-, +/+/+, +/-/+, and +/+/+ (IPTG/aTc/Ara), respectively. Source data are provided as a Source Data file.

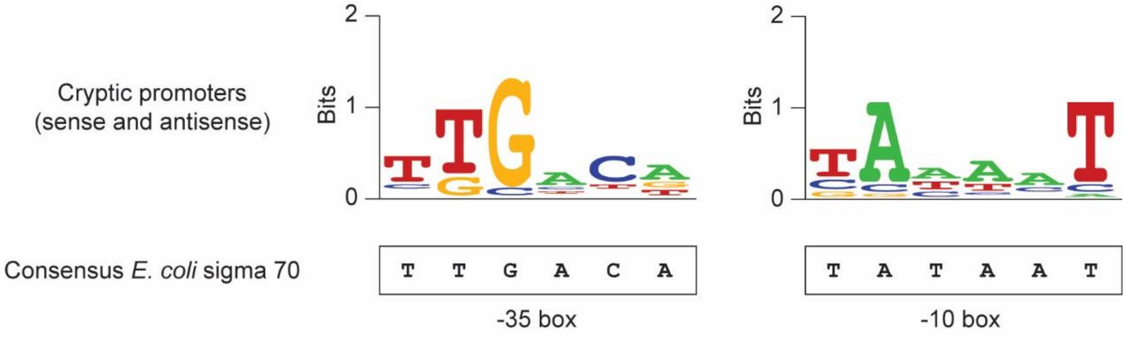

Supplementary Figure 8: **Identification of σ70 motifs in cryptic promoter sequences.** The consensus sites for -10 and -35 box of σ70 sequences are shown for 27 cryptic promoters with activity > 10^{-5} RNAP/s per DNA (Supplementary Table 1). Logos were generated using WebLogo[5]. Consensus *E. coli* sigma 70 motifs are also shown for comparison.

a

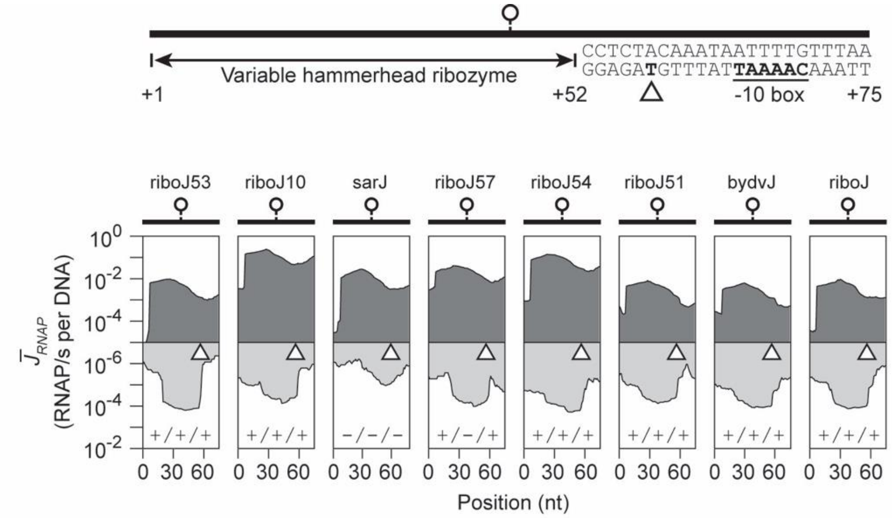

b

Gate	Ribozyme name	Ribozyme sequence
PhlF	riboJ53	AGCGGTCAACGCATGTGCTTTGCGTTCTGATGAGACAGTGATGTCGAAACCGCCTCT**A**CAAATAATTTTGTTTAA
SrpR	riboJ10	AGCGCTCAACGGGTGTGCTTCCCGTTCTGATGAGTCCGTGAGGACGAAAGCGCCTCT**A**CAAATAATTTTGTTTAA
BM3R1	sarJ	GACTGTCGCCGGATGTGTATCCGACCTGACGATGGCCCAAAAGGGCCGAAACAGTCCTCT**A**CAAATAATTTTGTTTAA
BetI	riboJ57	AGAAGTCAATTAATGTGCTTTTAATTCTGATGAGTCGGTGACGACGAAACTTCCTCT**A**CAAATAATTTTGTTTAA
AmeR	riboJ54	AGGGGTCAGTTGATGTGCTTTCAACTCTGATGAGTCAGTGATGACGAAACCCCCTCT**A**CAAATAATTTTGTTTAA
HlyIIR	riboJ51	AGTAGTCACCGGCTGTGCTTGCCGGTCTGATGAGCCTGTGAAGGCGAAACTACCTCT**A**CAAATAATTTTGTTTAA
AmtR	bydvJ	AGGGTGTCTCAAGGTGCGTACCTTGACTGATGAGTCCGAAAGGACGAAACACCCCTCT**A**CAAATAATTTTGTTTAA
YFP	riboJ	AGCTGTCACCGGATGTGCTTTCCGGTCTGATGAGTCCGTGAGGACGAAACAGCCTCT**A**CAAATAATTTTGTTTAA

Supplementary Figure 9: **Evidence for a cryptic antisense promoter in the ribozymes. (a)** A schematic is shown of the RiboJ class of insulators. The variable region is different for each insulator, but the sequence shown is the hairpin shared between them. The putative -10 box of the cryptic antisense promoter is indicated as well as the TSS (triangle). The RNA-seq profiles in the sense (top, dark grey) and antisense (bottom, light grey) are shown. The location of the TSSs are shown as triangles and these points correspond to an increase in transcription in the antisense direction. Data were selected for a particular combination of inducers, shown at the bottom of each graph (IPTG/aTc/Ara), but the evidence for the antisense promoter is present for all the states where the gene it is fused to is transcribed. **(b)** The full sequences of all ribozymes are shown with the constant hairpin sequence underlined, and the TSS of the cryptic antisense promoter in bold. Source data are provided as a Source Data file.

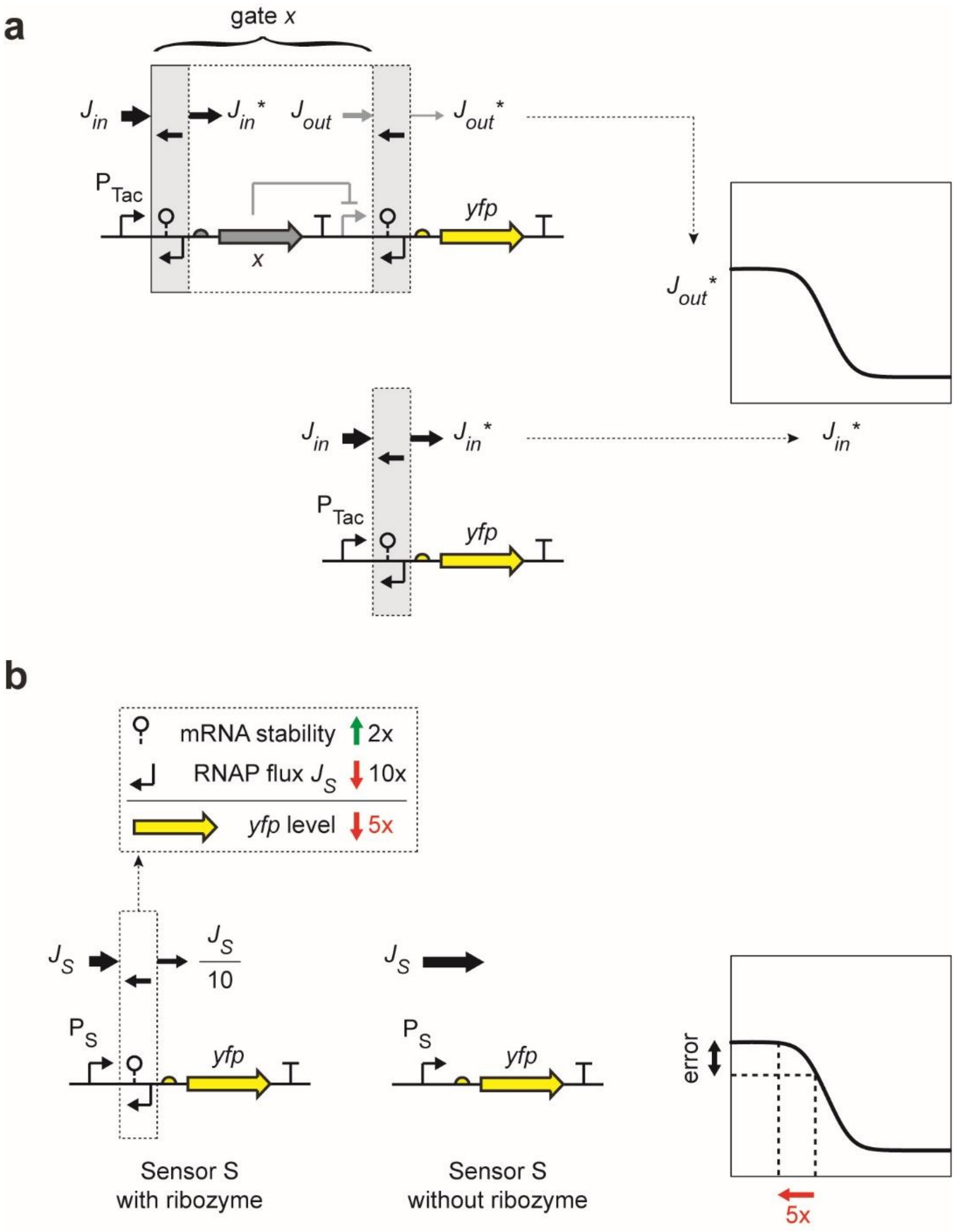

<u>Supplementary Figure 10:</u> **Importance of ribozyme in sensor and gate characterizations. (a)** The cryptic antisense promoters in the riboJ insulators pose a problem in defining the boundary across which the RNAP flux is quantified as an input to the gate. These antisense promoters act to reduce the "real" RNAP flux produced by the input promoter (that is the output promoter of a sensor or upstream gate). This effect was canceled out in our circuit design because the gates and all the sensors were characterized using riboJ upstream of the *yfp* reporter (and our reference promoter also uses riboJ). **(b)** A riboJ insulator controls the output of a sensor in two ways. It increases the mRNA stability by 2-fold[6], while its cryptic antisense promoter reduces the RNAP flux by around 10-fold, resulting in a 5-fold drop in sensor output activity. When connecting the sensor to a gate, with and without the ribozyme, this 5-fold difference in sensor output (input to the gate) can results in an error in the gate's output activity.

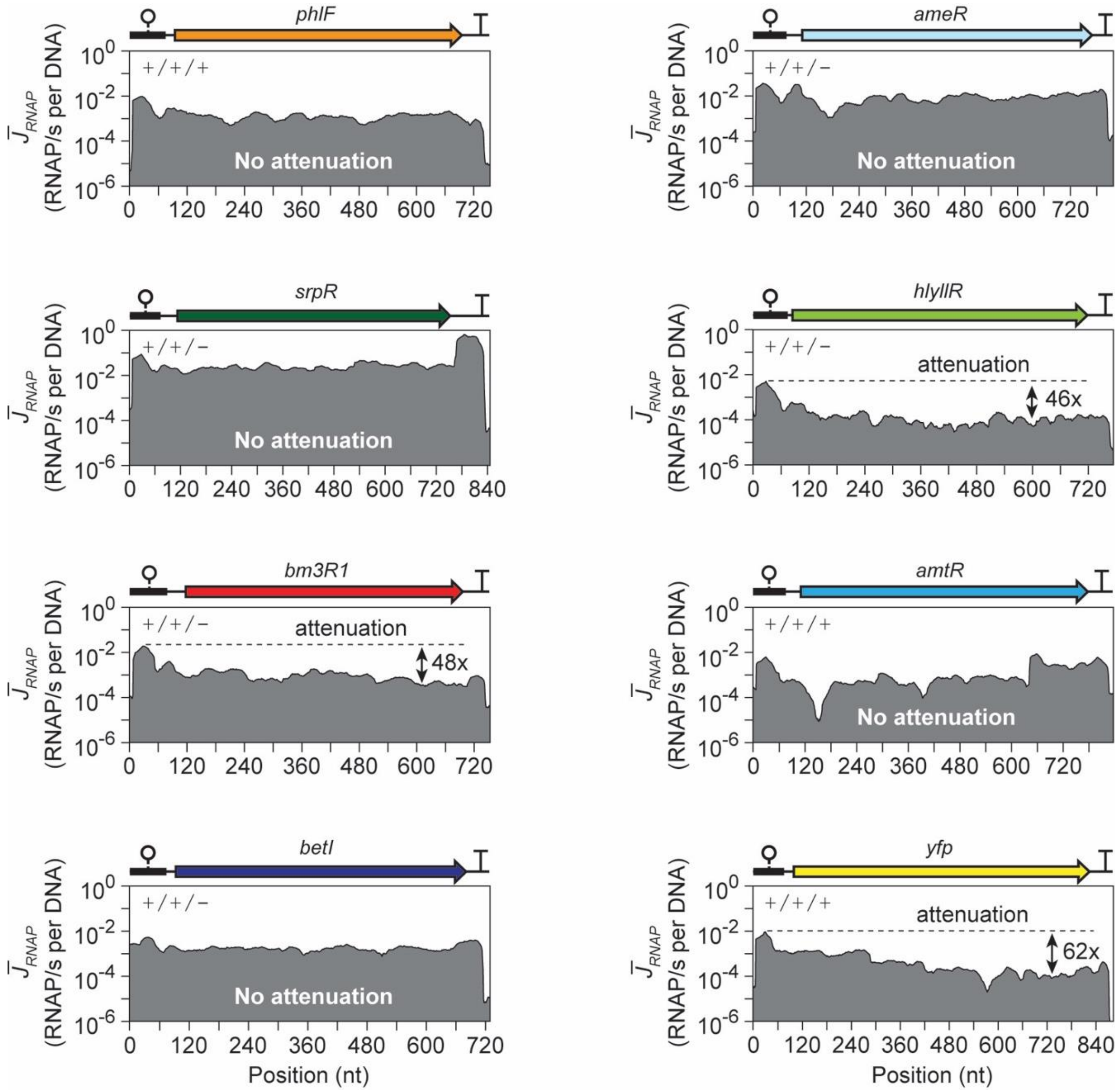

<u>Supplementary Figure 11:</u> **Evidence of transcriptional attenuation.** The RNA-seq profile is shown for the genes indicated at the top. The circuit state is shown in the upper left of each profile: IPTG/aTc/Ara. The dashed line is the maximum RNAP flux. When >10-fold continuous decrease in RNAP flux along the gene sequence is observed, this is interpreted as evidence for attenuation and is marked with the fold-change. Source data are provided as a Source Data file.

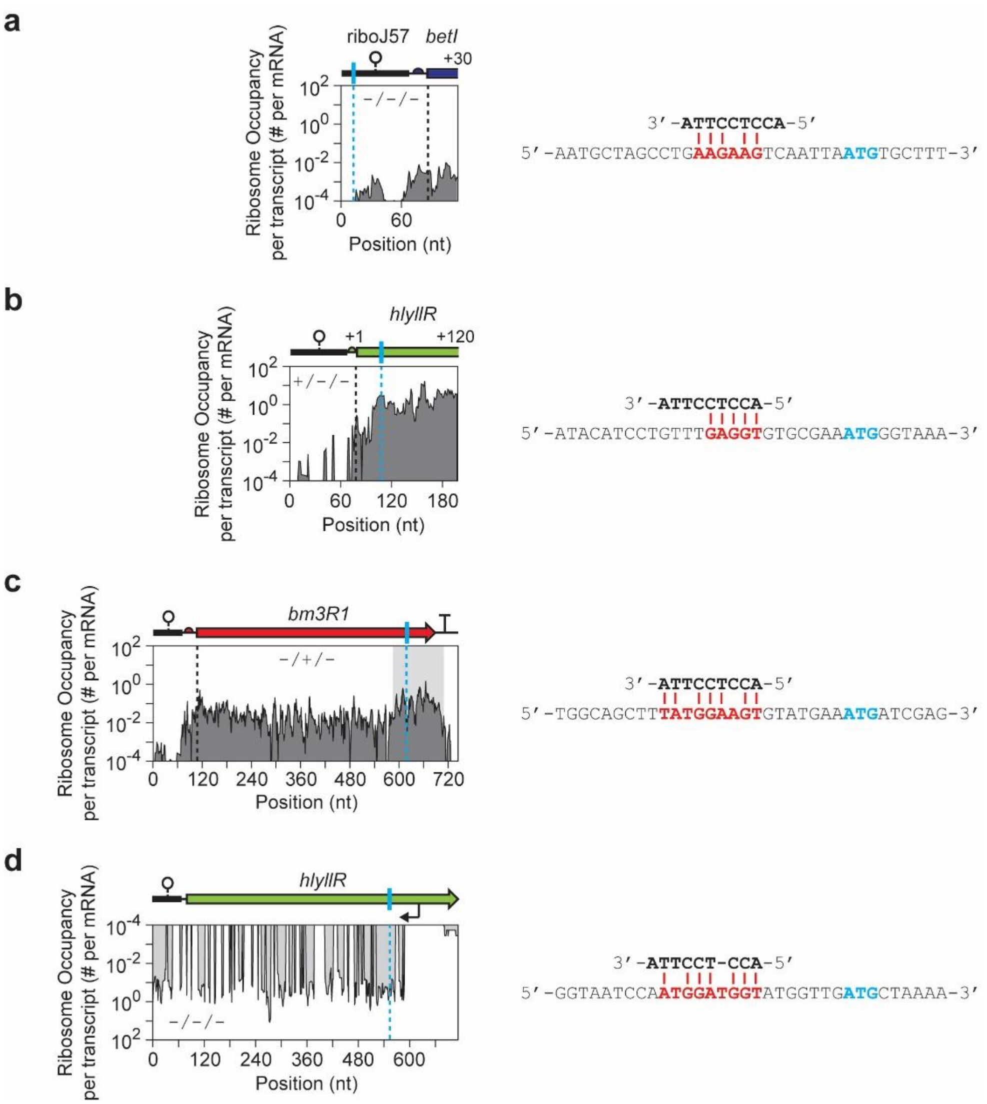

Supplementary Figure 12: **Evidence for translational errors.** Ribosome occupancy (per transcript) profiles demonstrate four different translational errors across the circuit. **(a)** Translation at the 5'-UTR. Black dashed lines are the annotated start codon for each gene and light blue dashed lines mark the position of start codons with upstream SD-like sequences found near the elevated part of the profiles. The inset shows the circuit state for which the data is shown: IPTG/aTc/Ara. To the right, the sequence corresponds to 25 nucleotides upstream of the start codon (light blue ATG) with the SD sequences highlighted in red. SD was identified as the region hybridizing with the 16S rRNA sequence (5'ACCTCCTTA3'), calculated using ViennaRNA software (version 1.8.5, default options)[7]. **(b)** Translation from alternative in-frame start codon. **(c)** Translation at internal start codon. **(d)** Translation from cryptic antisense transcript. Profile is shown in light grey and in downward orientation to indicate ribosome occupancy in antisense strand. The position of cryptic antisense promoter found in *hlyIIR* gene is shown by the reverse promoter symbol. The reverse-complement sequence in the right shows the start codon and SD-like sequences found on the antisense transcript upstream of that cryptic promoter. Source data are provided as a Source Data file.

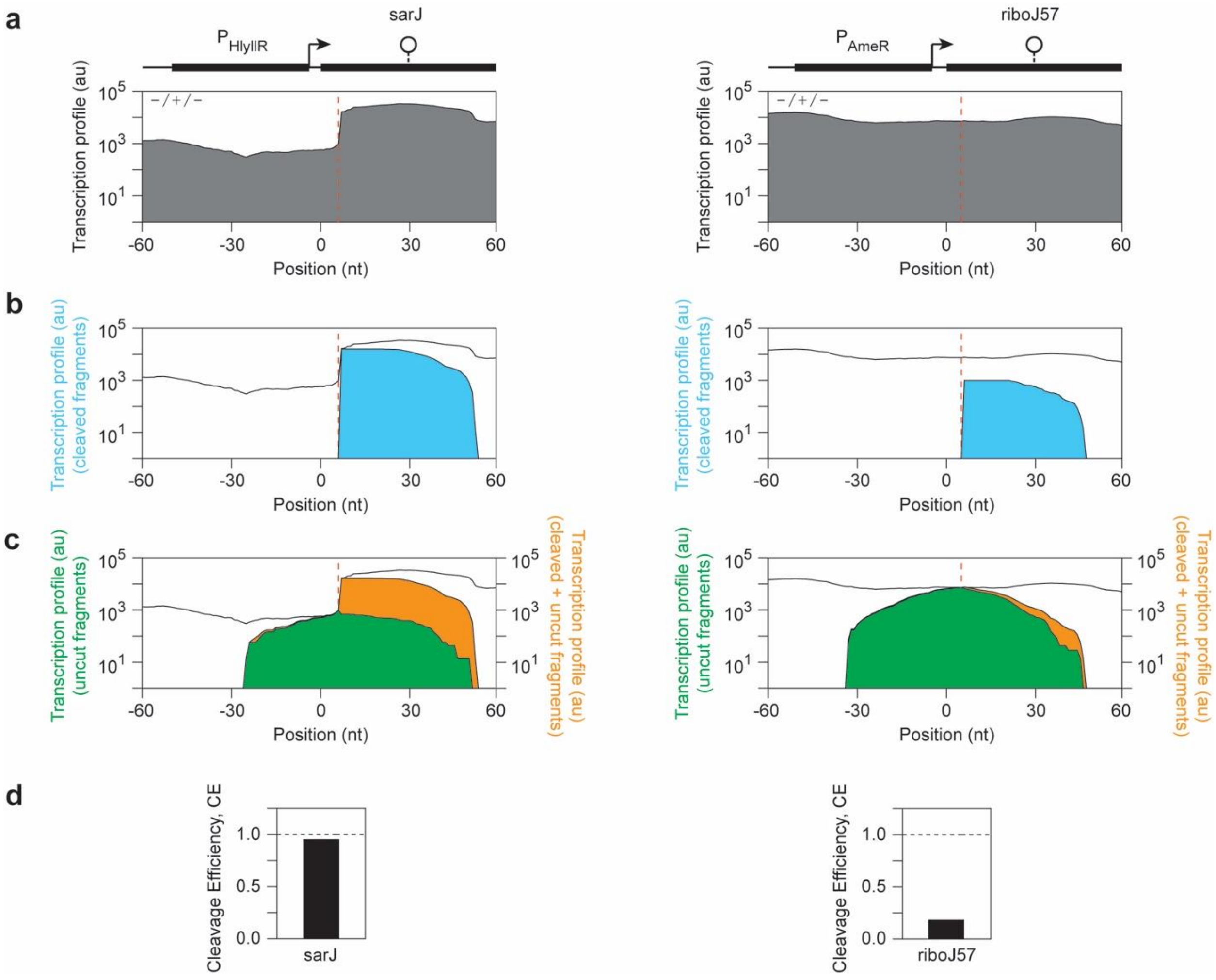

<u>Supplementary Figure 13:</u> **Calculation of ribozyme cleavage efficiency (CE). (a)** Transcription profiles around the sarJ (BM3R1 gate) and riboJ57 (BetI gate) ribozymes are shown for the circuit state -/+/- (IPTG/aTc/Ara). These profiles are the RNA-seq profiles normalized by the total mapped nucleotides, and are presented in arbitrary units (au). Red dashed lines denote the ribozyme cleavage sites. **(b)** Light blue regions show the pileup profile of mapped fragments that have been cut by ribozymes. **(c)** Green regions show the pileup profile of all uncut mapped fragments that span the cleavage site. Orange regions are the sum of two pileup profiles (cut (light blue) and uncut (green) fragments). **(d)** Cleavage efficiency (CE) of a ribozyme is calculated as the fraction of the cut fragments (light blue regions) to the sum of cut and uncut fragments (orange regions) downstream of the ribozyme cleavage site. Each bar is the value from one biological replicate. Source data are provided as a Source Data file.

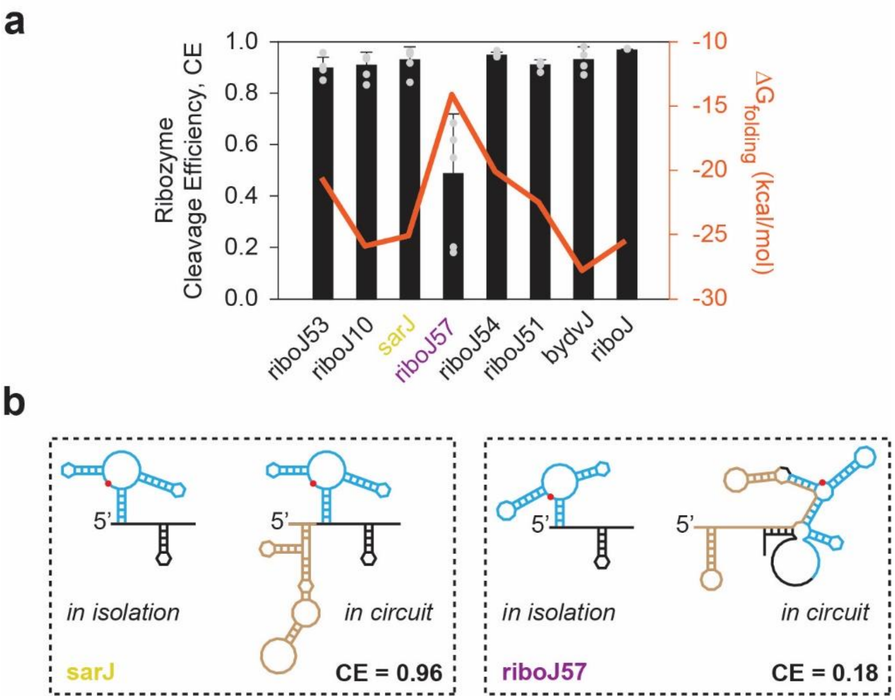

<u>Supplementary Figure 14:</u> Comparison of ribozyme cleavage efficiency (CE) and ∆G of RNA folding. (a) The measured ribozyme CE values are compared with the folding Gibbs free energy (∆G) of their corresponding hammerhead RNA structure (Methods). Grey dots shown for each ribozyme are the CE values across circuit states in which the promoters transcribing the ribozymes are on (the number of states (n) varies up to 6 across ribozymes). Black bars and their error bars show the average and standard deviation of individual dots for each ribozyme. **(b)** An example of mRNA secondary structure is shown for the best (sarJ) and worst (riboJ57) performing ribozymes using data from circuit state -/+/- (IPTG/aTc/Ara). Blue structures show the hammerhead RNA regions. Red dots are the ribozyme cleavage sites. Structures "in isolation" show the secondary structure of ribozyme alone, whereas structures "in circuit" show mRNA secondary structure when transcribed from upstream promoters in state -/+/-. Light brown structures are mRNA regions upstream of the ribozyme. For the sarJ ribozyme, the hammerhead RNA structure remains intact even in the circuit context. However, because hammerhead RNA structure in riboJ57 ribozyme is much weaker, ribozyme region undergoes conformational change when transcribed as a part of the long mRNA in the circuit, deactivating its cleavage capability. Source data are provided as a Source Data file.

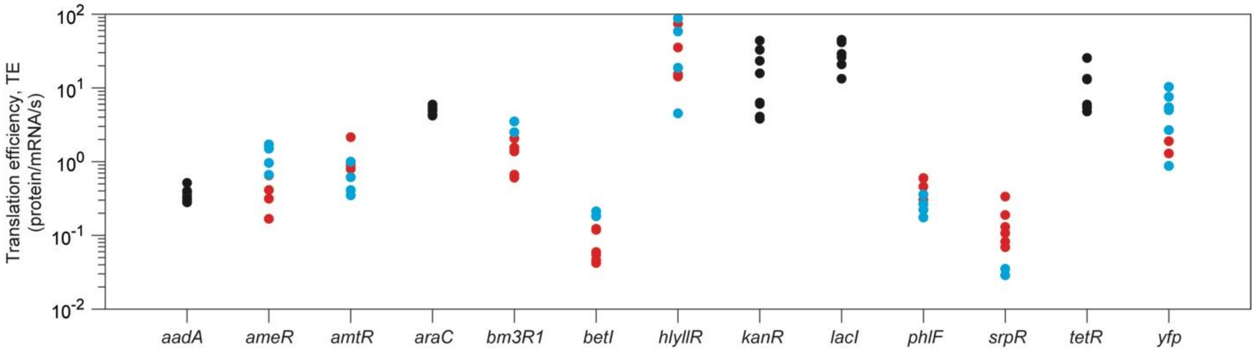

Supplementary Figure 15: The translation efficiency (TE) of genes across circuit states. Measured translation efficiencies (TE) in absolute units (protein/mRNA/s) of all 13 proteins in the circuit (3 sensors, 7 repressors, YFP, and 2 antibiotic markers) are shown. The circuit states are the eight combinations of inducers. When the gene on the x-axis is transcribed, the point is colored red (for repressors, the gate is off). When it is not transcribed, the point is blue (gates are on). Black dots are sensor proteins and antibiotic markers, which are transcribed for all combinations of inducers. Source data are provided as a Source Data file.

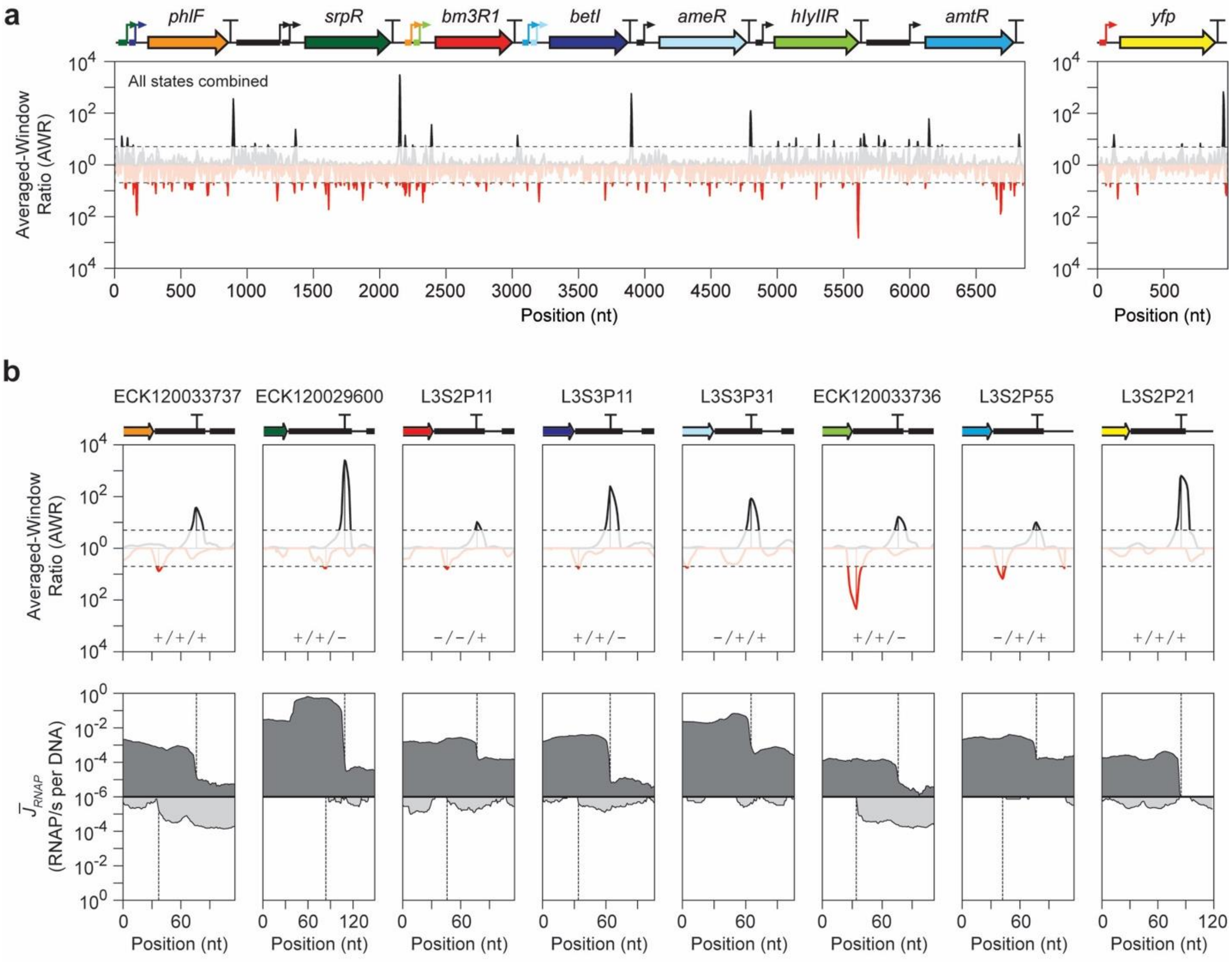

c

Gate	Terminator name	Terminator sequence
PhlF	ECK120033737	GGAAACACAGAAAAAAGCCCGCACCTGACAGTGCGGGCTTTTTTTTT**C**GACCAAAGG
SrpR	ECK120029600	TTCAGCCAAAAAACTTAAGACCGCCGGTCTTGTCCACTACCTTGCAGTAATGCGGTGGACAGGATCGGCGGTTTTCTTTT**C**TCTTCTCAA
BM3R1	L3S2P11	CTCGGTACCAAATTCCAGAAAAGAGACGCTTTCGAGCGTCTTTTTTCG**T**TTTGGTCC
BetI	L3S3P11	CCAATTATTGAACACCCTTCGGGGTGTTTTTTTGT**T**TCTGGTCTACC
AmeR	L3S3P31	CCAATTATTGAACACCCTAACGGGTGTTTTTTTTTT**T**TTGGTCTACC
HlyIIR	ECK120033736	AACGCATGAGAAAGCCCCCGGAAGATCACCTTCCGGGGGCTTTTTTA**T**TGCGC
AmtR	L3S2P55	CTCGGTACCAAAGACGAACAATAAGACGCTGAAAAGCGTCTTTTTTCG**T**TTTGGTCC
YFP	L3S2P21	CTCGGTACCAAATTCCAGAAAAGAGGCCTCCCGAAAGGGGGGCCTTTTTTCGT**T**TTGGTCC

Supplementary Figure 16: **Identification of the transcriptional termination site (TTS). (a)** The average-window ratio (AWR) profile (Methods) is shown on one graph for the circuit states corresponding to all eight combinations of inducers (eight profiles are overlaid on top of each other). The black and red lines correspond to the sense and antisense strands, respectively. The horizontal dashed lines are at AWR = 5. **(b)** The AWR profiles across all 8 terminators in the circuit (including 30 nucleotides before and after each terminator part) are shown for a particular combination of inducers, indicated at the bottom of each graph (IPTG/aTc/Ara). The bottom profiles are the corresponding RNAP

flux around each terminator at the same combination of inducers shown in AWR profiles. The dark and light gray regions correspond to the RNAP flux on the sense and antisense strands, respectively. Vertical lines depict the location of maximum AWR (the TTS). **(c)** The identified TTS's of all terminators used in the circuit are shown in bold. The underlined region corresponds to the poly-U terminator region. Source data are provided as a Source Data file.

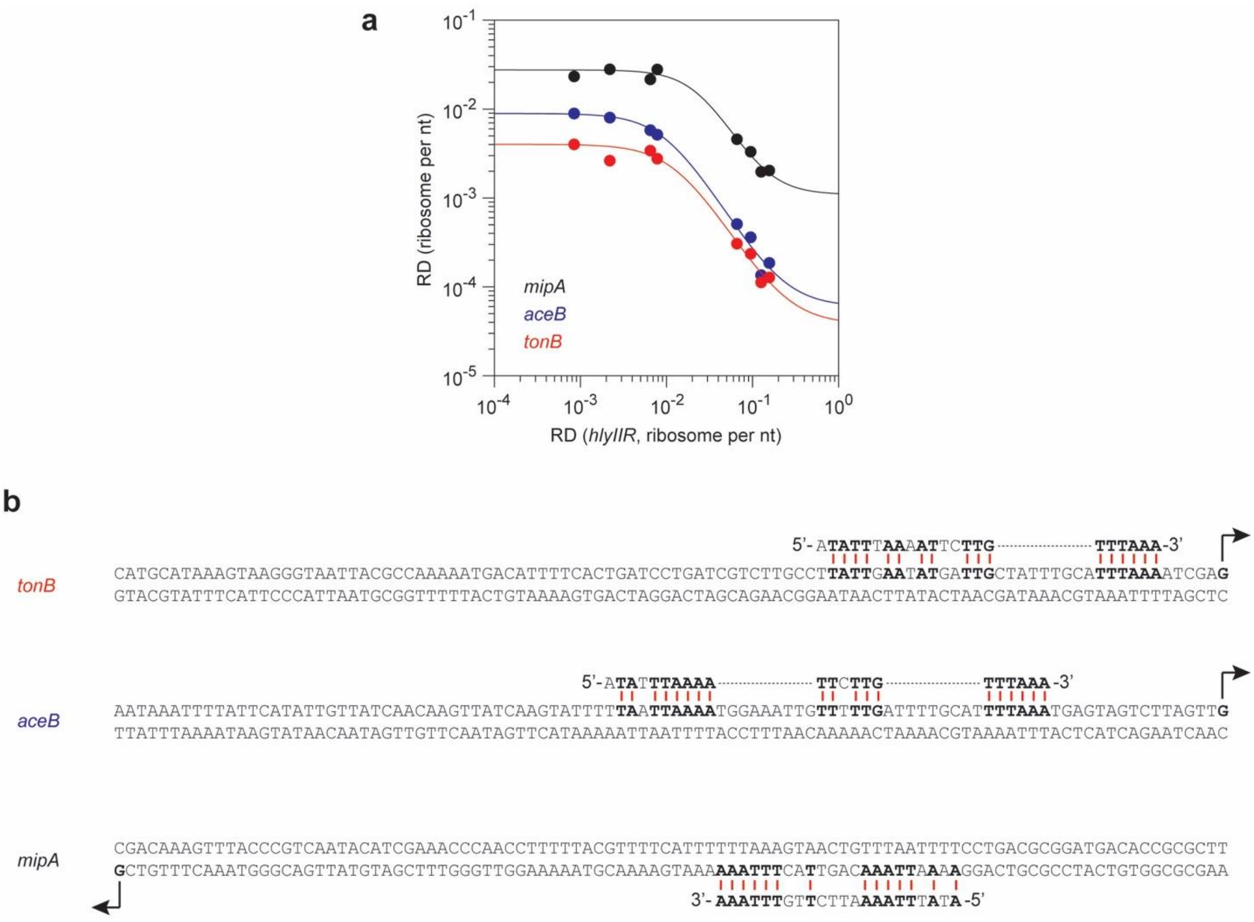

<u>Supplementary Figure 17:</u> Putative off-target HlyIIR binding sites in the genome. (a) Relationship between the ribosome density (RD) of the *hlyIIR* repressor and the RD of three native genes. The eight data points in each relationship are from the eight states of the circuit (combinations of inducers). The curves are best fits of the data to a Hill equation to guide the eye. **(b)** Sequences upstream of the annotated TSS of the off-target genes are shown. Bent arrows show the annotated TSS for each gene and the direction of transcription. The short DNA sequence is *hlyIIR*'s operator sequence[8], which was aligned to each sequence using Needleman–Wunsch algorithm[9] with mismatch penalty = 0, gap penalty = -2, and gap extension penalty = 0. Source data are provided as a Source Data file.

a

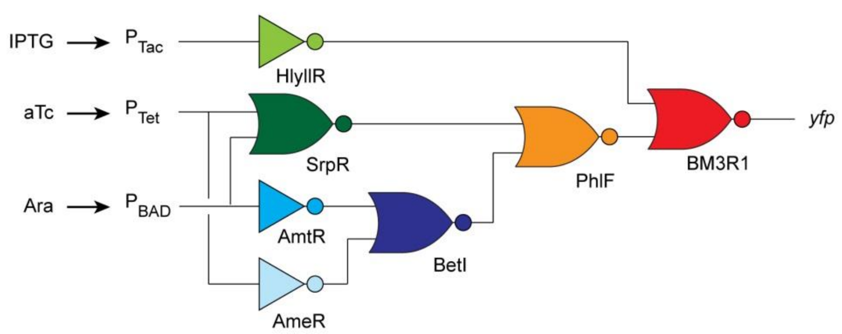

b

Expected truth table of the circuit

IPTG	aTc	Ara	P_{Tac}	P_{Tet}	P_{BAD}	P_{HlyIIR}	P_{SrpR}	P_{AmtR}	P_{AmeR}	P_{BetI}	P_{PhlF}	P_{BM3R1}	*yfp*
–	–	–	0	0	0	1	1	1	1	0	0	0	0
+	–	–	1	0	0	0	1	1	1	0	0	1	1
–	+	–	0	1	0	1	0	1	0	0	1	0	0
+	+	–	1	1	0	0	0	1	0	0	1	0	0
–	–	+	0	0	1	1	0	0	1	0	1	0	0
+	–	+	1	0	1	0	0	0	1	0	1	0	0
–	+	+	0	1	1	1	0	0	0	1	0	0	0
+	+	+	1	1	1	0	0	0	0	1	0	1	1

c

Truth table of circuit with failed gates

IPTG	aTc	Ara	P_{Tac}	P_{Tet}	P_{BAD}	P_{HlyIIR}	P_{SrpR}	P_{AmtR}	P_{AmeR}	P_{BetI}	P_{PhlF}	P_{BM3R1}	*yfp*
–	–	–	0	0	0	1	1	1	1	1	0	0	0
+	–	–	1	0	0	0	1	1	1	1	0	1	1
–	+	–	0	1	0	1	0	1	0	0	1	0	0
+	+	–	1	1	0	1	0	1	0	0	1	0	0
–	–	+	0	0	1	1	0	0	1	0	1	0	0
+	–	+	1	0	1	1	0	0	1	0	1	0	0
–	+	+	0	1	1	1	0	0	0	1	0	0	0
+	+	+	1	1	1	0	0	0	0	1	0	1	1

Supplementary Figure 18: **Boolean simulation of gate failures. (a)** The circuit diagram is shown with its gates labeled for reference. **(b)** The expected truth table of the circuit is shown for all 8 input states. The digits 0 and 1 represent when the promoters of a sensor or gate are off or on. The Boolean simulation simply takes the states of the inputs and propagates the signal through the circuit diagram. For example, if an input to a NOT gate is 1 then the output is 0 and vice versa. The on states of the *yfp* reporter represent the output of the circuit and they are highlighted with yellow when in state 1. **(c)** The same Boolean simulation is performed as in part b, except the HlyIIR and BetI gates fail in the same way that they do empirically (shown in red). With all these failed gates, the circuit still generates the correct output *yfp*. In other words, the final columns of parts b and c match despite the failures in the latter.

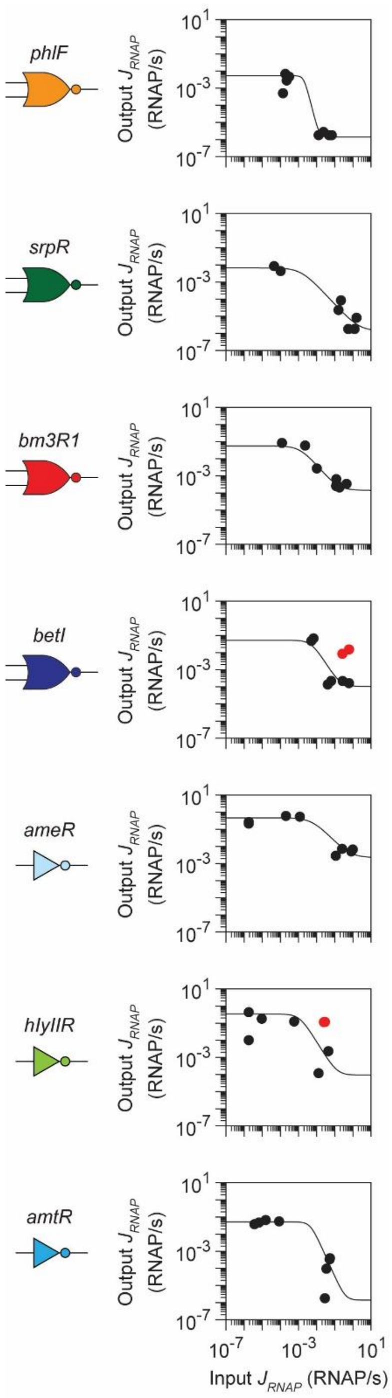

Supplementary Figure 19: **Fitting the gate response functions to omics data.** Input and output promoter activity data for each gate is shown across all eight circuit states (combinations of inducers). Points in red represent states of BetI and HlyIIR gates that had failed. These outliers were disregarded when calculating the best fit for these two gates. The line indicates the best fit to the data (Equation 1), with fitted parameters *K* (in units of RNAP/s) and *n* (dimensionless). The fitted *K* is then converted to the repressor binding constant *k* in units of protein number using Equation 22. The resulting repressor binding constant *k* and *n* are presented in Table 2. Source data are provided as a Source Data file.

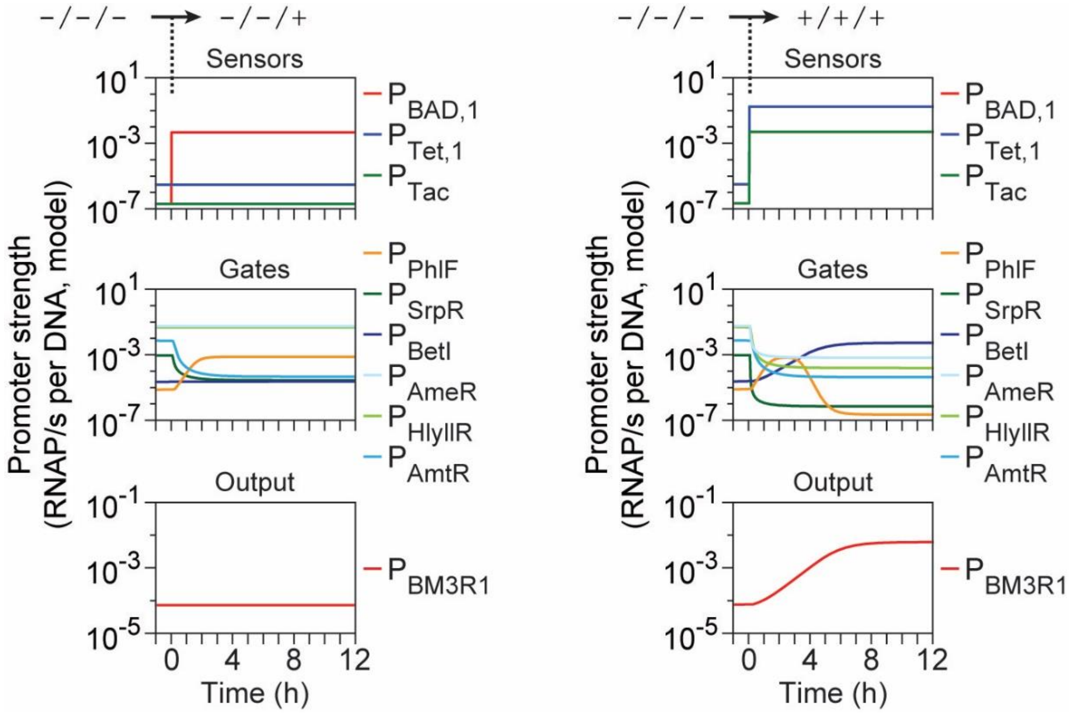

<u>Supplementary Figure 20:</u> Dynamic modelling of genetic circuit using parameters extracted from omics data. See Methods for simulation details. These graphs are showing transitions between states, expanding on the one shown in Figure 5a. The switch between induction conditions is shown at the top (IPTG/aTc/Ara). The top panel indicates the changes in the sensor promoter activity, with $P_{BAD,2}$ and $P_{Tet,2}$ not shown as they are similar to $P_{BAD,1}$ and $P_{Tet,1}$, respectively. In both simulations, the transition in the activity of output promoter (P_{BM3R1}) is monotonic, and circuit is free of any glitches. Source data are provided as a Source Data file.

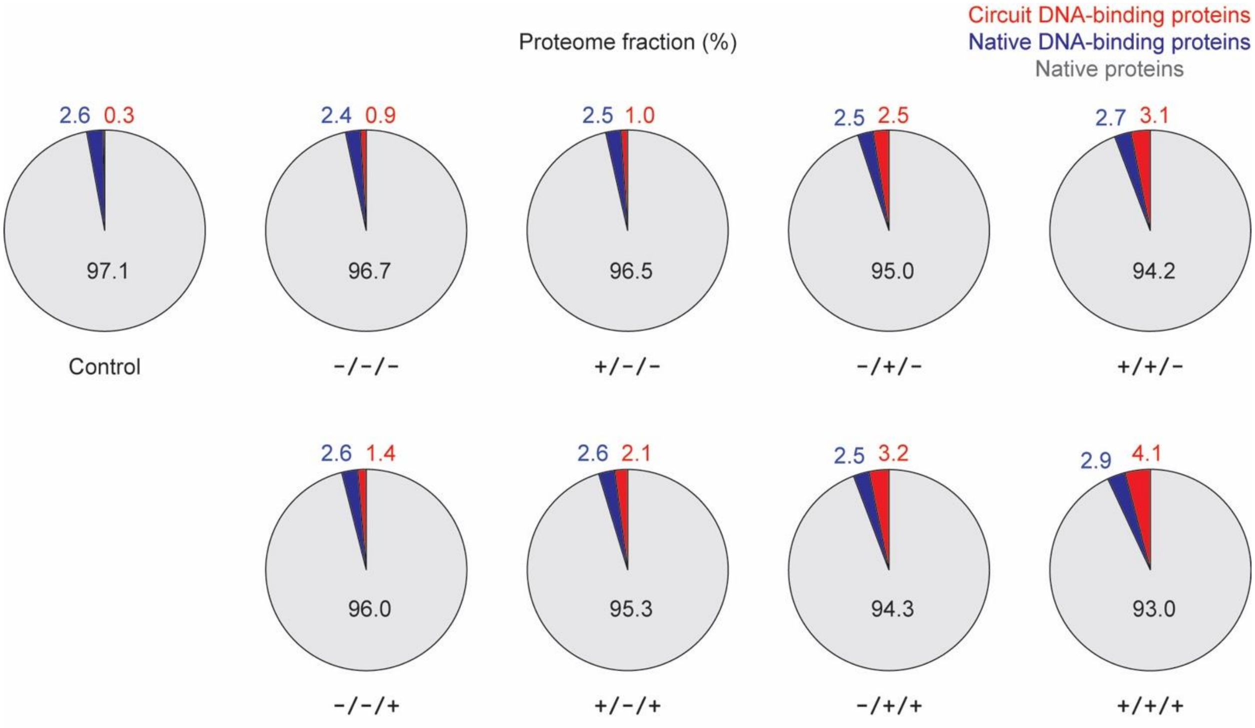

Supplementary Figure 21: **Estimated proteome fraction of the circuit and native DNA-binding proteins.** The proteome fraction of circuit DNA-binding proteins (7 repressors, 3 sensors) (red) is compared with the proteome fraction of 283 DNA-binding proteins in *E. coli* DH10B (blue) across all circuit induction states (IPTG/aTc/Ara). Grey is the proteome fraction of all other native proteins in *E. coli* DH10B. Note that these proteome fractions are the estimates of the actual proteome fractions in the circuit and genome. Source data are provided as a Source Data file.

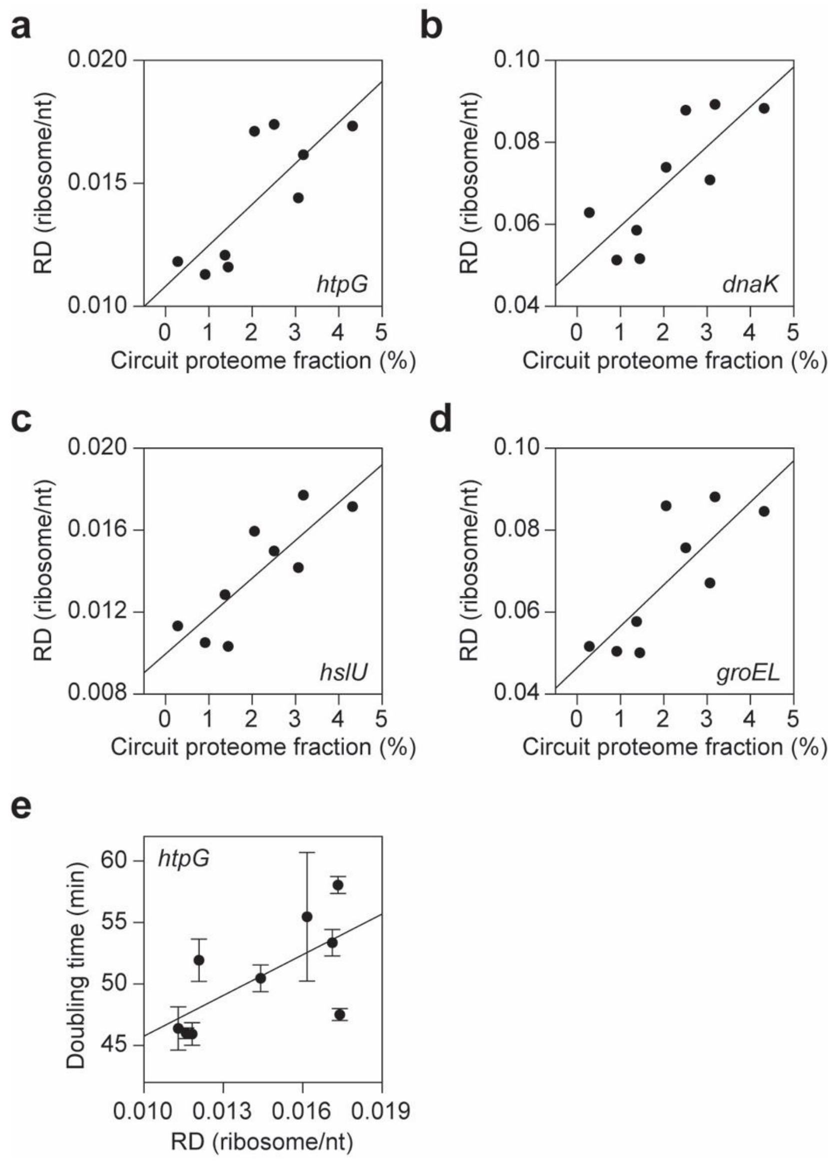

<u>Supplementary Figure 22:</u> **Expression of major chaperons in *E. coli* as a function of circuit proteome fraction. (a to d)** Ribosome densities (RD) of four major protein folding chaperons in *E. coli* are shown against circuit proteome fraction. The nine data points in each plot represent the eight induction states of the circuit as well as the control cell which has the lowest proteome fraction (0.3%). Lines are the best fits. (R^2, p-value) = (0.62, 0.012) **(a)**, (0.63, 0.010) **(b)**, (0.70, 0.005) **(c)**, and (0.63, 0.010) **(d)**. **(e)** Average cell doubling times +/- standard deviations (measured on three different days, n = 3) is shown against the RD of *htpG* protein folding chaperon for 8 induction states of the circuit and the control cell. The line is a best fit to a linear equation (R^2 = 0.44, p-value = 0.051). Note that these RD and proteome fractions are the estimates of the actual protein expression levels in the circuit and genome. Source data are provided as a Source Data file.

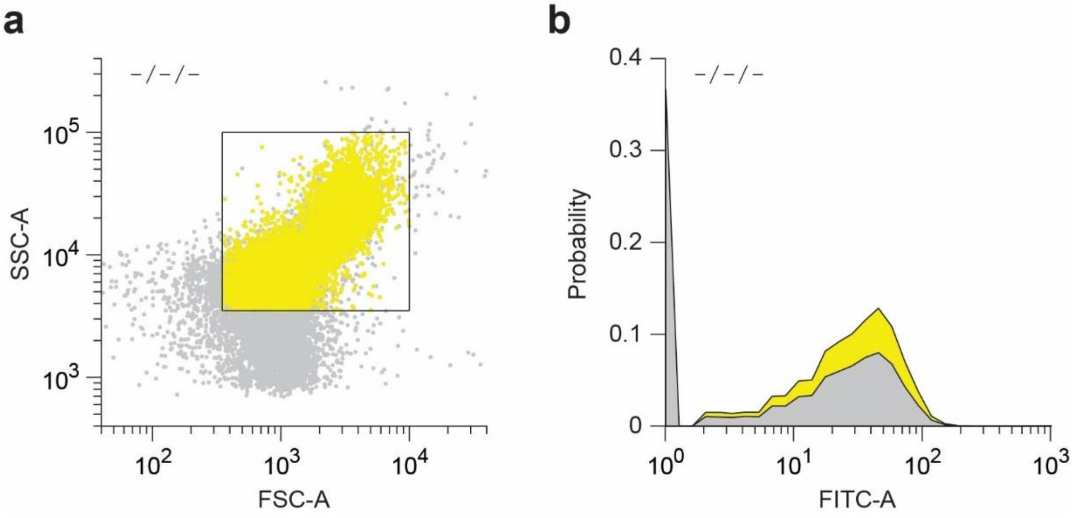

<u>Supplementary Figure 23:</u> An example of gated flow cytometry data. (a) The raw light scatter data (grey color, 30837 events) are gated to select the events associated with *E. coli* cells (350 < FSC-A < 10000 and 3500 < SSC-A < 100000, yellow color, 17580 events). SSC-A and FSC-A are the side-scattered and forward-scattered lights for each event, respectively. In addition, the gated events all have positive *yfp* fluorescence values (FITC-A > 0). Data are shown for the circuit induction state -/-/- (IPTG/aTc/Ara). **(b)** The distribution of fluorescence values are shown for all the raw (grey) and gated (yellow) events, shown in **a**. The gating removes the raw events with very low fluorescence values. Source data are provided as a Source Data file.

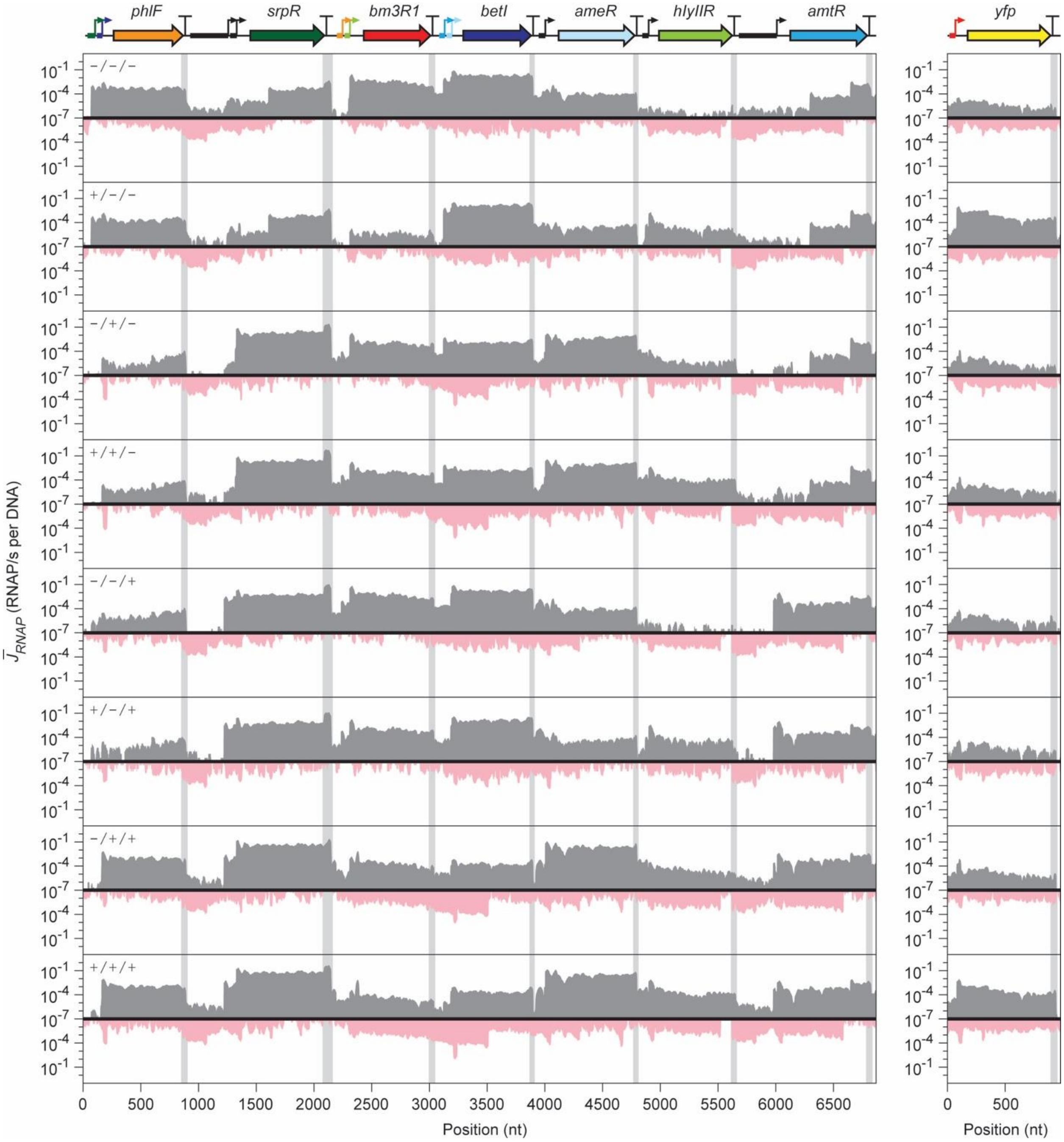

Supplementary Figure 24: **Sense and antisense transcription profiles.** RNAP flux profile in absolute units (RNAP/s per DNA) is shown on both the sense (grey) and antisense (pink) strands of the circuit for all eight induction states (shown in the upper left of each graph): IPTG/aTc/Ara. Grey shaded bars represent terminator regions. Source data are provided as a Source Data file.

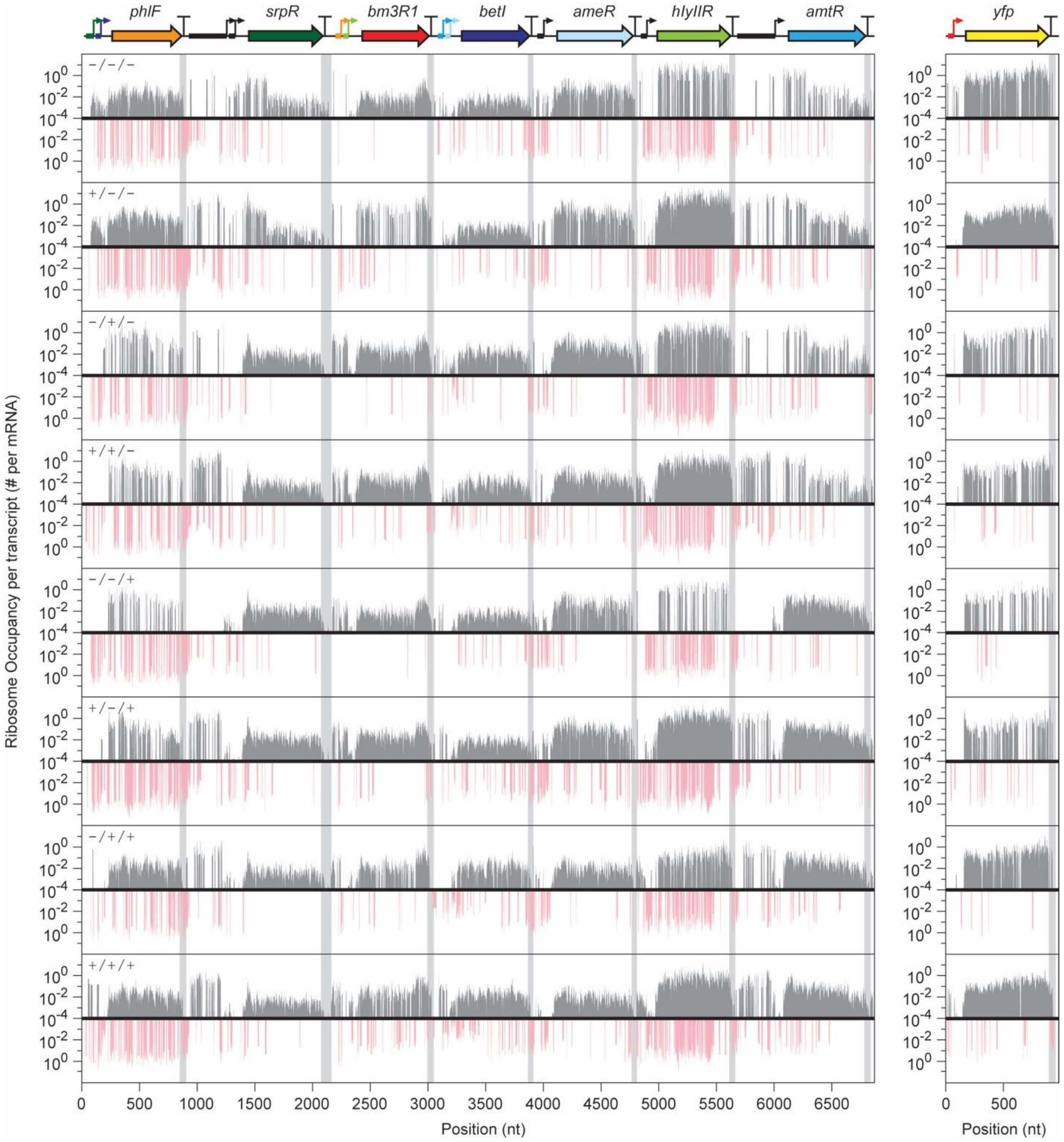

Supplementary Figure 25: Ribosome occupancy per transcript on both sense and antisense strands. Ribosome occupancy per transcript in absolute units (ribosome per mRNA) (Methods) is shown on both the sense (grey) and antisense (pink) strands of the circuit for all 8 induction states. Grey shaded bars represent terminator regions. The circuit state is shown in the upper left of each graph: IPTG/aTc/Ara. These data correspond to Figure 1d, where only the sense occupancies are shown. Source data are provided as a Source Data file.

Supplementary Table 1: **Sequences of cryptic sense and antisense promoters.**

Position[a]	Direction	Sequence[b]
1976	Sense	CAGATTCGTTACCAATTGACAGCTAGCTCAGTCCTAGGTATATACATACA
2503	Sense	GGCGTGAAACCATTTGTGGTGAAGCATTTCGTTGTGTTATTGCAGAAGCA
3128	Sense	TTAGCGGATCCTACCTGACGCTTTTTATCGCAACTCTCTACTGTTTCTCC
3515	Sense	GTGTTACCCGTGGTGCAGTTTATTGGCATTTTAATGGTAAACTGGAAGTT
5828	Sense	AATTATTGAACACCCTTCGGGGTGTTTTTTTGTTTCTGGTCTACCGTAAT
6778	Sense	CGATCGTTGGCTGTGTTGACAATTAATCATCGGCTCGTATAATGTGTGGA
7887	Sense	TTAGCGGATCCTACCTGACGCTTTTTATCGCAACTCTCTACTGTTTCTCC
8203	Sense	AGATTGCAGATGCAGTTGGTATTCGTCAGGCAAGCCTGTATTATCATTTT
8556	Sense	ATTACCATGAGCGTTATTGAAATGCGTCGCAATGATGGTAAAATTCCGAG
2120	Antisense	GGGGTACGTGCCATAGGATCGCCCTCGTAAAGTTAAACAAAATTATTTGT
2985	Antisense	GCAAATAATCAATGTGGACTTTTCTGCCGTGATTATAGACACTTTTGTTA
3288	Antisense	GTATCTCATATGTGAAAACATAGTCCATAGACTCTTAAACAAAATTATTT
5153	Antisense	CCCAGTTTCGGCATGTGCTACTCCTCGGGGGGTTAAACAAAATTATTTGT
5423	Antisense	TTACCACCAACAATTGCCTGCAGACGCTGTTCTGCGCTACCCTGCGGCAG
5453	Antisense	GCTGCGCTGCTAACCTGGGTTTCATCAAAATTACCACCAACAATTGCCTG
5521	Antisense	CCTGCTGCAGACGATACAGCATCGGCTGATGCATGCTGCTTGCCCAAAAT
5593	Antisense	CTTGTGCCTGTTCACGAGGCAGTTCACGACGAAATTCGCTAACCAGATTG
6214	Antisense	CCAAAACCAATTGCATCAACCAGTGCGTTTTTGCTGCTAAAATGTTTAAA
6228	Antisense	AAACACCAATCTGACCAAAACCAATTGCATCAACCAGTGCGTTTTTGCTG
6589	Antisense	AGATCAACCAGCTGATCACAACGGGTTGCCAGATTACCAATATCTTCTTG
6600	Antisense	CTGCATCAATCAGATCAACCAGCTGATCACAACGGGTTGCCAGATTACCA
6859	Antisense	ACCTCAAACAGGATGTATTTCATCTCGGGGGTTTAAACAAAATTATTTGT
7434	Antisense	GGCTAACCAGATCTTCCGGCATCCATTCATGATTGGTATCATTGGTTTCA
7744	Antisense	GCAAATAATCAATGTGGACTTTTCTGCCGTGATTATAGACACTTTTGTTA
8498	Antisense	TTACCATCATTGCGACGCATTTCAATAACGCTCATGGTAATATGAAACGG
1080*	Antisense	CACCATCTAGTATTTCCCCTCTTTCTCTAGTATTAAACAAAATTATTTGT
1277*	Antisense	CCGTAGCCGAAGGTGGTCACGAGGGTGGGCCAGGGCACGGGCAGCTTGCC

[a] *The spacing follows the position of the promoter's TSS in the circuit or reporter (labeled by asterisk) plasmids.*

[b] *All promoter sequences are shown 5' to 3' (TSS). The sequences that have similarity to σ70 -10 box (TATAAT) and -35 box (TTGACA) are highlighted in red (see also Supplementary Figure 8).*

Supplementary Table 2: **Genes upregulated by higher resource usage.**

Gene	Pathway	Pathway ID	Correlation (R^2)[a]
ugd	colanic acid biosynthetic process	GO:0009242	0.64
cpsB	colanic acid biosynthetic process	GO:0009242	0.61
cysP	sulfur compound metabolic process	GO:0006790	0.54
cysP	sulfate transport	GO:0008272	0.54
wzc	colanic acid biosynthetic process	GO:0009242	0.52
lamB	maltodextrin transport	GO:0042956	0.46
malK	maltodextrin transport	GO:0042956	0.44
cysU	sulfate transport	GO:0008272	0.37
cysD	sulfur compound metabolic process	GO:0006790	0.36
cysK	cysteine biosynthetic process from serine	GO:0006535	0.34
cysN	sulfur compound metabolic process	GO:0006790	0.28

[a] The correlations between RD of the target gene and the proteome fraction occupied by circuit genes (for all eight circuit states).

Supplementary Table 3: Genes downregulated by higher resource usage.

Gene	Pathway	Pathway ID	Correlation (R^2)[a]
sdhC	aerobic respiration	GO:0009060	0.88
sucA	tricarboxylic acid cycle	GO:0006099	0.84
mdh	tricarboxylic acid cycle	GO:0006099	0.84
sucB	tricarboxylic acid cycle	GO:0006099	0.82
sdhB	aerobic respiration	GO:0009060	0.82
sdhA	tricarboxylic acid cycle	GO:0006099	0.81
sdhA	aerobic respiration	GO:0009060	0.81
sucC	tricarboxylic acid cycle	GO:0006099	0.81
sucD	tricarboxylic acid cycle	GO:0006099	0.81
betI	response to osmotic stress	GO:0006970	0.80
sdhD	aerobic respiration	GO:0009060	0.80
acnB	tricarboxylic acid cycle	GO:0006099	0.79
lldD	aerobic respiration	GO:0009060	0.70
cyoB	aerobic respiration	GO:0009060	0.62
cyoC	aerobic respiration	GO:0009060	0.58
cyoA	aerobic respiration	GO:0009060	0.57
otsA	trehalose biosynthetic process	GO:0005992	0.55
otsA	response to osmotic stress	GO:0006970	0.55
fumA	tricarboxylic acid cycle	GO:0006099	0.52
otsB	trehalose biosynthetic process	GO:0005992	0.43
otsB	response to osmotic stress	GO:0006970	0.43
cyoD	aerobic respiration	GO:0009060	0.43

[a] The correlations between RD of the target gene and the proteome fraction occupied by circuit genes (for all eight circuit states).

Supplementary Table 4: **Plasmid sequences.**

Plasmid name	sequence[a]
pDV4-P_{BAD}-yfp	ATGAGGGAAGCGGTGATCGCCGAAGTATCGACTCAACTATCAGAGGTAGTTGGCGTCATCGAGCGCCATCTCGAACCGACGTTGCTGGCCGT<br>ACATTTGTACGGCTCCGCAGTGGATGGCGGCCTGAAGCCACACAGTGATATTGATTTGCTGGTTACGGTGACCGTAAGGCTTGATGAAACAA<br>CGCGGCGAGCTTTGATCAACGACCTTTTGGAAACTTCGGCTTCCCCTGGAGAGAGCGAGATTCTCCGCGCTGTAGAAGTCACCATTGTTGTG<br>CACGACGACATCATTCCGTGGCGTTATCCAGCTAAGCGCGAACTGCAATTTGGAGAATGGCAGCGCAATGACATTCTTGCAGGTATCTTCGA<br>GCCAGCCACGATCGACATTGATCTGGCTATCTTGCTGACAAAAGCAAGAGAACATAGCGTTGCCTTGGTAGGTCCAGCGGCGGAGGAACTCT<br>TTGATCCGGTTCCTGAACAGGATCTATTTGAGGCGCTAAATGAAACCTTAACGCTATGGAACTCGCCGCCCGACTGGGCTGGCGATGAGCGA<br>AATGTAGTGCTTACGTTGTCCCGCATTTGGTACAGCGCAGTAACCGGCAAAATCGCGCCGAAGGATGTCGCTGCCGACTGGGCAATGGAGCG<br>CCTGCCGGCCCAGTATCAGCCCGTCATACTTGAAGCTAGACAGGCTTATCTTGGACAAGAAGAAGATCGCTTGGCCTCGCGCGCAGATCAGT<br>TGGAAGAATTTGTCCACTACGTGAAAGGCGAGATCACCAAGGTAGTCGGCAAATAATACTAGCTCCGGCAAAAAAACGGGCAAGGTGTCACC<br>ACCCTGCCCTTTTTCTTTAAAACCGAAAAGATTACTTCGCGTTTGCCACCTGACGTCTAAGAACTCGGTACCAAACCAATTATTGAAGACGC<br>TGAAAAGCGTCTTTTTTTGTTTCGGTCCGCTTACTTTTCATACTCCCGCCATTCAGAGAAGAAACCAATTGTCCATATTGCATCAGACATTG<br>CCGTCACTGCGTCTTTTACTGGCTCTTCTCGCTAACCAAACCGGTAACCCCGCTTATTAAAAGCATTCTGTAACAAAGCGGGACCAAAGCCA<br>TGACAAAAACGCGTAACAAAAGTGTCTATAATCACGGCAGAAAAGTCCACATTGATTATTTGCACGGCGTCACACTTTGCTATGCCATAGCA<br>TTTTTATCCATAAGATTAGCGGATCCTACCTGACGCTTTTTATCGCAACTCTCTACTGTTTCTCCATACCCGTTTTTTTGGGCTAGCCTGAA<br>GCTGTCACCGGATGTGCTTTCCGGTCTGATGAGTCCGTGAGGACGAAACAGCCTCTACAAATAATTTTGTTTAATACTAGAGAAAGAGGGGA<br>AATACTAGATGGTGAGCAAGGGCGAGGAGCTGTTCACCGGGGTGGTGCCCATCCTGGTCGAGCTGGACGGCGACGTAAACGGCCACAAGTTC<br>AGCGTGTCCGGCGAGGGCGAGGGCGATGCCACCTACGGCAAGCTGACCCTGAAGTTCATCTGCACCACAGGCAAGCTGCCCGTGCCCTGGCC<br>CACCCTCGTGACCACCTTCGGCTACGGCCTGCAATGCTTCGCCCGCTACCCCGACCACATGAAGCTGCACGACTTCTTCAAGTCCGCCATGC<br>CCGAAGGCTACGTCCAGGAGCGCACCATCTTCTTCAAGGACGACGGCAACTACAAGACCCGCGCCGAGGTGAAGTTCGAGGGCGACACCCTG<br>GTGAACCGCATCGAGCTGAAGGGCATCGACTTCAAGGAGGACGGCAACATCCTGGGGCACAAGCTGGAGTACAACTACAACAGCCACAACGT<br>CTATATCATGGCCGACAAGCAGAAGAACGGCATCAAGGTGAACTTCAAGATCCGCCACAACATCGAGGACGGCAGCGTGCAGCTCGCCGACC<br>ACTACCAGCAGAACACCCCAATCGGCGACGGCCCCGTGCTGCTGCCCGACAACCACTACCTTAGCTACCAGTCCGCCCTGAGCAAAGACCCC<br>AACGAGAAGCGCGATCACATGGTCCTGCTGGAGTTCGTGACCGCCGCCGGGATCACTCTCGGCATGGACGAGCTGTACAAGTAACTCGGTAC<br>CAAATTCCAGAAAAGAGGCCTCCCGAAAGGGGGGCCTTTTTTCGTTTTGGTCCGCTCACTCAAAGGCGGTAATACGGTTATCCACAGAATCA<br>GGGGATAACGCAGGAAAGAACATGTGAGCAAAAGGCCAGCAAAAGGCCAGGAACCGTAAAAACTGTCAGACCAAGTTTACGAGCTCGCTTGG<br>ACTCCTGTTGATAGATCCAGTAATGACCTCAGAACTCCATCTGGATTTGTTCAGAACGCTCGGTTGCCGCCGGGCGTTTTTTATTGGTGAGA<br>ATCCAAGCACTAGGGACAGTAAGACGGGTAAGCCTGTTGATGATACCGCTGCCTTACTGGGTGCATTAGCCAGTCTGAATGACCTGTCACGG<br>GATAATCCGAAGTGGTCAGACTGGAAAATCAGAGGGCAGGAACTGCTGAACAGCAAAAAGTCAGATAGCACCACATAGCAGACCCGCCATAA<br>AACGCCCTGAGAAGCCCGTGACGGGCTTTTCTTGTATTATGGGTAGTTTCCTTGCATGAATCCATAAAAGGCGCCTGTAGTGCCATTTACCC<br>CCATTCACTGCCAGAGCCGTGAGCGCAGCGAACTGAATGTCACGAAAAAGACAGCGACTCAGGTGCCTGATGGTCGGAGACAAAAGGAATAT<br>TCAGCGATTTGCCCGAGCTTGCGAGGGTGCTACTTAAGCCTTTAGGGTTTTAAGGTCTGTTTTGTAGAGGAGCAAACAGCGTTTGCGACATC<br>CTTTTGTAATACTGCGGAACTGACTAAAGTAGTGAGTTATACACAGGGCTGGGATCTATTCTTTTTATCTTTTTTTATTCTTTCTTTATTCT<br>ATAAATTATAACCACTTGAATATAAACAAAAAAAACACACAAAGGTCTAGCGGAATTTACAGAGGGTCTAGCAGAATTTACAAGTTTTCCAG<br>CAAAGGTCTAGCAGAATTTACAGATACCCACAACTCAAAGGAAAAGGACATGTAATTATCATTGACTAGCCCATCTCAATTGGTATAGTGAT<br>TAAAATCACCTAGACCAATTGAGATGTATGTCTGAATTAGTTGTTTTCAAAGCAAATGAACTAGCGATTAGTCGCTATGACTTAACGGAGCA<br>TGAAACCAAGCTAATTTTATGCTGTGTGGCACTACTCAACCCCACGATTGAAAACCCTACAAGGAAAGAACGGACGGTATCGTTCACTTATA<br>ACCAATACGCTCAGATGATGAACATCAGTAGGGAAAATGCTTATGGTGTATTAGCTAAAGCAACCAGAGAGCTGATGACGAGAACTGTGGAA<br>ATCAGGAATCCTTTGGTTAAAGGCTTTGAGATTTTCCAGTGGACAAACTATGCCAAGTTCTCAAGCGAAAAATTAGAATTAGTTTTTAGTGA<br>AGAGATATTGCCTTATCTTTTCCAGTTAAAAAAATTCATAAAATATAATCTGGAACATGTTAAGTCTTTTGAAAACAAATACTCTATGAGGA<br>TTTATGAGTGGTTATTAAAAGAACTAACACAAAAGAAAACTCACAAGGCAAATATAGAGATTAGCCTTGATGAATTTAAGTTCATGTTAATG<br>CTTGAAAATAACTACCATGAGTTTAAAAGGCTTAACCAATGGGTTTTGAAACCAATAAGTAAAGATTTAAACACTTACAGCAATATGAAATT<br>GGTGGTTGATAAGCGAGGCCGCCCGACTGATACGTTGATTTTCCAAGTTGAACTAGATAGACAAATGGATCTCGTAACCGAACTTGAGAACA<br>ACCAGATAAAAATGAATGGTGACAAAATACCAACAACCATTACATCAGATTCCTACCTACGTAACGGACTAAGAAAAACACTACACGATGCT<br>TTAACTGCAAAAATTCAGCTCACCAGTTTTGAGGCAAAATTTTTGAGTGACATGCAAAGTAAGCATGATCTCAATGGTTCGTTCTCATGGCT<br>CACGCAAAAACAACGAACCACACTAGAGAACATACTGGCTAAATACGGAAGGATCTGAGGTTCTTATGGCTCTTGTATCTATCAGTGAAGCA<br>TCAAGACTAACAAACAAAAGTAGAACAACTGTTCACCGTTAGATATCAAAGGGAAAACTGTCCATATGCACAGATGAAAACGGTGTAAAAAA<br>GATAGATACATCAGAGCTTTTACGAGTTTTTGGTGCATTTAAAGCTGTTCACCATGAACAGATCGACAATGTAACGATTATCAAAAAGGATC<br>TTCACCTAGATCCTTTTAAATTAAAAATGAAGCTTCGTTGTGTCTCAAAATCTCTGATGTTACATTGCACAAGATAAAAATATATCATCATG<br>AACAATAAAACTGTCTGCTTACATAAACAGTAATACAAGGGGTGTTTACTAGAGGTTGATCGGGCACGTAAGAGGTTCCAACTTTCACCATA<br>ATGAAATAAGATCACTACCGGGCGTATTTTTTGAGTTATCGAGATTTTCAGGAGCTAAGGAAGCTAAA
pHlyIIR-H1-ΔATG	GCTTCCTCGCTCACTGACTCGCTGCACGAGGCAGACCTCAGCGCTAGCGGAGTGTATACTGGCTTACTATGTTGGCACTGATGAGGGTGTCA<br>GTGAAGTGCTTCATGTGGCAGGAGAAAAAAGGCTGCACCGGTGCGTCAGCAGAATATGTGATACAGGATATATTCCGCTTCCTCGCTCACTG<br>ACTCGCTACGCTCGGTCGTTCGACTGCGGCGAGCGGAAATGGCTTACGAACGGGGCGGAGATTTCCTGGAAGATGCCAGGAAGATACTTAAC<br>AGGGAAGTGAGAGGGCCGCGGCAAAGCCGTTTTTCCATAGGCTCCGCCCCCCTGACAAGCATCACGAAATCTGACGCTCAAATCAGTGGTGG<br>CGAAACCCGACAGGACTATAAAGATACCAGGCGTTTCCCCTGGCGGCTCCCTCGTGCGCTCTCCTGTTCCTGCCTTTCGGTTTACCGGTGTC<br>ATTCCGCTGTTATGGCCGCGTTTGTCTCATTCCACGCCTGACACTCAGTTCCGGGTAGGCAGTTCGCTCCAAGCTGGACTGTATGCACGAAC<br>CCCCCGTTCAGTCCGACCGCTGCGCCTTATCCGGTAACTATCGTCTTGAGTCCAACCCGGAAAGACATGCAAAAGCACCACTGGCAGCAGCC<br>ACTGGTAATTGATTTAGAGGAGTTAGTCTTGAAGTCATGCGCCGGTTAAGGCTAAACTGAAAGGACAAGTTTTGGTGACTGCGCTCCTCCAA<br>GCCAGTTACCTCGGTTCAAAGAGTTGGTAGCTCAGAGAACCTTCGAAAAACCGCCCTGCAAGGCGGTTTTTTCGTTTTCAGAGCAAGAGATT<br>ACGCGCAGACCAAAACGATCTCAAGAAGATCATCTTATTAAGGGGTCTGACGCTCAGTGGAACGAAAAATCAATCTAAAGTATATATGAGTA<br>AACTTGGTCTGACAGTTACCTTAGAAAAACTCATCGAGCATCAAATGAAACTGCAATTTATTCATATCAGGATTATCAATACCATATTTTTG<br>AAAAAGCCGTTTCTGTAATGAAGGAGAAAACTCACCGAGGCAGTTCCATAGGATGGCAAGATCCTGGTATCGGTCTGCGATTCCGACTCGTC<br>CAACATCAATACAACCTATTAATTTCCCCTCGTCAAAAATAAGGTTATCAAGTGAGAAATCACCATGAGTGACGACTGAATCCGGTGAGAAT<br>GGCAAAAGCTTATGCATTTCTTTCCAGACTTGTTCAACAGGCCAGCCATTACGCTCGTCATCAAAATCACTCGCATCAACCAAACCGTTATT<br>CATTCGTGATTGCGCCTGAGCGAGACGAAATACGCGATCGCTGTTAAAAGGACAATTACAAACAGGAATCGAATGCAACCGGCGCAGGAACA<br>CTGCCAGCGCATCAACAATATTTTCACCTGAATCAGGATATTCTTCTAATACCTGGAATGCTGTTTTCCCGGGGATCGCAGTGGTGAGTAAC<br>CATGCATCATCAGGAGTACGGATAAAATGCTTGATGGTCGGAAGAGGCATAAATTCCGTCAGCCAGTTTAGTCTGACCATCTCATCTGTAAC<br>ATCATTGGCAACGCTACCTTTGCCATGTTTCAGAAACAACTCTGGCGCATCGGGCTTCCCATACAATCGATAGATTGTCGCACCTGATTGCC<br>CGACATTATCGCGAGCCCATTTATACCCATATAAATCAGCATCCATGTTGGAATTTAATCGCGGCCTCGAGCAAGACGTTTCCCGTTGAATA<br>TGGCTCATAACACCCCTTGTATTACTGTTTATGTAAGCAGACAGTTTTATTGTTCATGATGATATATTTTTATCTTGTGCAATGTACATCAG<br>AGATTTTGAGACACAACCAATTATTGAAGGCCTCCCTAACGGGGGGCCTTTTTTTGTTTCTGGTCTCCCGCTTAACGATCGTTGGCTGTGTT

GACAATTAATCATCGGCTCGTATAATGTGTGGAATTGTGAGCGCTCACAATTCTGAAGTAGTCACCGGCTGTGCTTGCCGGTCTGATGAGCC
TGTGAAGGCGAAACTACCTCTACAAATAATTTTGTTTAAACCCCCGAGAAATACATCCTGTTTGAGGTGTGCGAAATGGGTAAAAGCCGTGA
ACAGACCATGGAAAATATTCTGAAAGCAGCCAAAAAGAAATTCGGCGAACGTGGTTATGAAGGCACCAGCATTCAAGAAATTACCAAAGAAG
CCAAAGTTAACGTTGCAATGGCCAGCTATTACTTTAATGGCAAAGAGAACCTGTACTACGAGGTGTTCAAAAAATACGGTCTGGCAAATGAA
CTGCCGAACTTTCTGGAAAAAAACCAGTTTAATCCGATTAATGCCCTGCGTGAATATCTGACCGTTTTTACCACCCACATTAAAGAAAATCC
GGAAATTGGCACCCTGGCCTATGAAGAAATTATCAAAGAAAGCGCACGCCTGGAAAAAATCAAACCGTATTTTATCGGCAGCTTCGAACAGC
TGAAAGAAATTCTGCAAGAGGGTGAAAAACAGGGTGTGTTTCACTTTTTTAGCATCAACCATACCATCCATTGGATTACCAGCATTGTTCTG
TTTCCGAAATTCAAAAAATTCATCGATAGCCTGGGTCCGAATGAAACCAATGATACCAATCATGAATGGATGCCGGAAGATCTGGTTAGCCG
TATTATTAGCGCACTGACCGATAAACCGAACATTTAAAACGCATGAGAAAGCCCCCGGAAGATCACCTTCCGGGGGCTTTTTTATTGCGCAA
TGACCAGGAATCTGAACGATTCGTTACCAATTGACATATTTAAAATTCTTGTTTAAAATGCTAGCCTGAAGCTGTCACCGGATGTGCTTTCC
GGTCTGATGAGTCCGTGAGGACGAAACAGCCTCTACAAATAATTTTGTTTAATACTAGAGAAAGAGGGGAAATACTAGATGGTGAGCAAGGG
CGAGGAGCTGTTCACCGGGGTGGTGCCCATCCTGGTCGAGCTGGACGGCGACGTAAACGGCCACAAGTTCAGCGTGTCCGGCGAGGGCGAGG
GCGATGCCACCTACGGCAAGCTGACCCTGAAGTTCATCTGCACCACAGGCAAGCTGCCCGTGCCCTGGCCCACCCTCGTGACCACCTTCGGC
TACGGCCTGCAATGCTTCGCCCGCTACCCCGACCACATGAAGCTGCACGACTTCTTCAAGTCCGCCATGCCCGAAGGCTACGTCCAGGAGCG
CACCATCTTCTTCAAGGACGACGGCAACTACAAGACCCGCGCCGAGGTGAAGTTCGAGGGCGACACCCTGGTGAACCGCATCGAGCTGAAGG
GCATCGACTTCAAGGAGGACGGCAACATCCTGGGGCACAAGCTGGAGTACAACTACAACAGCCACAACGTCTATATCATGGCCGACAAGCAG
AAGAACGGCATCAAGGTGAACTTCAAGATCCGCCACAACATCGAGGACGGCAGCGTGCAGCTCGCCGACCACTACCAGCAGAACACCCCAAT
CGGCGACGGCCCCGTGCTGCTGCCCGACAACCACTACCTTAGCTACCAGTCCGCCCTGAGCAAAGACCCCAACGAGAAGCGCGATCACATGG
TCCTGCTGGAGTTCGTGACCGCCGCCGGGATCACTCTCGGCATGGACGAGCTGTACAAGTAACTCGGTACCAAATTCCAGAAAAGAGGCCTC
CCGAAAGGGGGGCCTTTTTTCGTTTTGGTCCAATGGCGGCGCGCCATCGAATGGCGCAAAACCTTTCGCGGTATGGCATGATAGCGCCCGGA
AGAGAGTCAATTCAGGGTGGTGAATATGAAACCAGTAACGTTATACGATGTCGCAGAGTATGCCGGTGTCTCTTATCAGACCGTTTCCCGCG
TGGTGAACCAGGCCAGCCACGTTTCTGCGAAAACGCGGGAAAAAGTGGAAGCGGCGATGGCGGAGCTGAATTACATTCCCAACCGCGTGGCA
CAACAACTGGCGGGCAAACAGTCGTTGCTGATTGGCGTTGCCACCTCCAGTCTGGCCCTGCACGCGCCGTCGCAAATTGTCGCGGCGATTAA
ATCTCGCGCCGATCAACTGGGTGCCAGCGTGGTGGTGTCGATGGTAGAACGAAGCGGCGTCGAAGCCTGTAAAGCGGCGGTGCACAATCTTC
TCGCGCAACGCGTCAGTGGGCTGATCATTAACTATCCGCTGGATGACCAGGATGCCATTGCTGTGGAAGCTGCCTGCACTAATGTTCCGGCG
TTATTTCTTGATGTCTCTGACCAGACACCCATCAACAGTATTATTTTCTCCCATGAGGACGGTACGCGACTGGGCGTGGAGCATCTGGTCGC
ATTGGGTCACCAGCAAATCGCGCTGTTAGCGGGCCCATTAAGTTCTGTCTCGGCGCGTCTGCGTCTGGCTGGCTGGCATAAATATCTCACTC
GCAATCAAATTCAGCCGATAGCGGAACGGGAAGGCGACTGGAGTGCCATGTCCGGTTTTCAACAAACCATGCAAATGCTGAATGAGGGCATC
GTTCCCACTGCGATGCTGGTTGCCAACGATCAGATGGCGCTGGGCGCAATGCGCGCCATTACCGAGTCCGGGCTGCGCGTTGGTGCGGATAT
CTCGGTAGTGGGATACGACGATACCGAAGATAGCTCATGTTATATCCCGCCGTTAACCACCATCAAACAGGATTTTCGCCTGCTGGGGCAAA
CCAGCGTGGACCGCTTGCTGCAACTCTCTCAGGGCCAGGCGGTGAAGGGCAATCAGCTGTTGCCAGTCTCACTGGTGAAAAGAAAAACCACC
CTGGCGCCCAATACGCAAACCGCCTCTCCCCGCGCGTTGGCCGATTCATTAATGCAGCTGGCACGACAGGTTTCCCGACTGGAAAGCGGGCA
GTGATAATCCAGGAGGAAAAAAATGTCCAGATTAGATAAAAGTAAAGTGATTAACAGCGCATTAGAGCTGCTTAATGAGGTCGGAATCGAAG
GTTTAACAACCCGTAAACTCGCCCAGAAGCTAGGTGTAGAGCAGCCTACATTGTATTGGCATGTAAAAAATAAGCGGGCTTTGCTCGACGCC
TTAGCCATTGAGATGTTAGATAGGCACCATACTCACTTTTGCCCTTTAGAAGGGGAAAGCTGGCAAGATTTTTTACGTAATAACGCTAAAAG
TTTTAGATGTGCTTTACTAAGTCATCGCGATGGAGCAAAAGTACATTTAGGTACACGGCCTACAGAAAAACAGTATGAAACTCTCGAAAATC
AATTAGCCTTTTTATGCCAACAAGGTTTTTCACTAGAGAATGCATTATATGCACTCAGCGCTGTGGGGCATTTTACTTTAGGTTGCGTATTG
GAAGATCAAGAGCATCAAGTCGCTAAAGAAGAAAGGGAAACACCTACTACTGATAGTATGCCGCCATTATTACGACAAGCTATCGAATTATT
TGATCACCAAGGTGCAGAGCCAGCCTTCTTATTCGGCCTTGAATTGATCATATGCGGATTAGAAAAACAACTTAAATGTGAAAGTGGGTCCT
AATAATTGGTAACGAATCAGACAATTGACGGCTCGAGGGAGTAGCATAGGGTTTGCAGAATCCCTGCTTCGTCCATTTGACAGGCACATTAT
GCATCGATGATAAGCTGTCAAACATGAGCAGATCCTCTACGCCGGACGCATCGTGGCCGGCATCACCGGCGCCACAGGTGCGGTTGCTGGCG
CCTATATCGCCGACATCACCGATGGGGAAGATCGGGCTCGCCACTTCGGGCTCATGAGCAAATATTTTATCTGAGGT

[a] *Yellow highlight is the YFP transcription unit (including ribozyme, RBS, yfp, and terminator). Light blue highlight is the P_{BAD} promoter. Green highlight is the mutated HlyIIR transcription unit (including ribozyme, RBS, mutated hlyIIR (ΔATG), and terminator). Coding section of hlyIIR without the ATG(+1) is underlined. The in-frame ATG inside hlyIIR coding section is highlighted in red. Grey highlight is the P_{HlyIIR} promoter transcribing yfp.*

Supplementary Table 5: **Genetic part sequences.**

Part name	Type	DNA Sequence
BBa_J23101	Promoter	TTTACAGCTAGCTCAGTCCTAGGTATTATGCTAGC
BBa_J23105	Promoter	TTTACGGCTAGCTCAGTCCTAGGTACTATGCTAGC
P_{SrpR}	Promoter	TCTATGATTGGTCCAGATTCGTTACCAATTGACAGCTAGCTCAGTCCTAGGTATATACATACATGCTTGTTTG TTTGTAAAC
P_{BetI}	Promoter	AGCGCGGGTGAGAGGGATTCGTTACCAATTGACAATTGATTGGACGTTCAATATAATGCTAGC
$P_{BAD,1}$	Promoter	ACTTTTCATACTCCCGCCATTCAGAGAAGAAACCAATTGTCCATATTGCATCAGACATTGCCGTCACTGCGTC TTTTACTGGCTCTTCTCGCTAACCAAACCGGTAACCCCGCTTATTAAAAGCATTCTGTAACAAAGCGGGACCA AAGCCATGACAAAAACGCGTAACAAAAGTGTCTATAATCACGGCAGAAAAGTCCACATTGATTATTTGCACGG CGTCACACTTTGCTATGCCATAGCATTTTTATCCATAAGATTAGCGGATCCTACCTGACGCTTTTTATCGCAA CTCTCTACTGTTTCTCCATACCCGTTTTTTTGGGCTAGC
$P_{Tet,1}$	Promoter	TACTCCACCGTTGGCTTTTTTCCCTATCAGTGATAGAGATTGACATCCCTATCAGTGATAGAGATAATGAGCA C
P_{PhlF}	Promoter	CGACGTACGGTGGAATCTGATTCGTTACCAATTGACATGATACGAAACGTACCGTATCGTTAAGGT
P_{HlyIIR}	Promoter	ACCAGGAATCTGAACGATTCGTTACCAATTGACATATTTAAAATTCTTGTTTAAAATGCTAGC
P_{AmtR}	Promoter	CTTGTCCAACCAAATGATTCGTTACCAATTGACAGTTTCTATCGATCTATAGATAATGCTAGC
P_{AmeR}	Promoter	TCGTCACTAGAGGGCGATAGTGACAAACTTGACAACTCATCACTTCCTAGGTATAATGCTAGC
$P_{Tet,2}$	Promoter	TACTCCACCGTTGGCTTTTTTCCCTATCAGTGATAGAGATTGACATCCCTATCAGTGATAGAGATAATGAGCA C
P_{Tac}	Promoter	AACGATCGTTGGCTGTGTTGACAATTAATCATCGGCTCGTATAATGTGTGGAATTGTGAGCGCTCACAATT
$P_{BAD,2}$	Promoter	ACTTTTCATACTCCCGCCATTCAGAGAAGAAACCAATTGTCCATATTGCATCAGACATTGCCGTCACTGCGTC TTTTACTGGCTCTTCTCGCTAACCAAACCGGTAACCCCGCTTATTAAAAGCATTCTGTAACAAAGCGGGACCA AAGCCATGACAAAAACGCGTAACAAAAGTGTCTATAATCACGGCAGAAAAGTCCACATTGATTATTTGCACGG CGTCACACTTTGCTATGCCATAGCATTTTTATCCATAAGATTAGCGGATCCTACCTGACGCTTTTTATCGCAA CTCTCTACTGTTTCTCCATACCCGTTTTTTTGGGCTAGC
P_{BM3R1}	Promoter	AATCCGCGTGATAGGTCTGATTCGTTACCAATTGACGGAATGAACGTTCATTCCGATAATGCTAGC
RiboJ53	Insulator	AGCGGTCAACGCATGTGCTTTGCGTTCTGATGAGACAGTGATGTCGAAACCGCCTCTACAAATAATTTTGTTT AA
RiboJ10	Insulator	AGCGCTCAACGGGTGTGCTTCCCGTTCTGATGAGTCCGTGAGGACGAAAGCGCCTCTACAAATAATTTTGTTT AA
SarJ	Insulator	GACTGTCGCCGGATGTGTATCCGACCTGACGATGGCCCAAAAGGGCCGAAACAGTCCTCTACAAATAATTTTG TTTAA
RiboJ57	Insulator	AGAAGTCAATTAATGTGCTTTTAATTCTGATGAGTCGGTGACGACGAAACTTCCTCTACAAATAATTTTGTTT AA
RiboJ54	Insulator	AGGGGTCAGTTGATGTGCTTTCAACTCTGATGAGTCAGTGATGACGAAACCCCCTCTACAAATAATTTTGTTT AA
RiboJ51	Insulator	AGTAGTCACCGGCTGTGCTTGCCGGTCTGATGAGCCTGTGAAGGCGAAACTACCTCTACAAATAATTTTGTTT AA
BydvJ	Insulator	AGGGTGTCTCAAGGTGCGTACCTTGACTGATGAGTCCGAAAGGACGAAACACCCCTCTACAAATAATTTTGTT TAA

RiboJ	Insulator	AGCTGTCACCGGATGTGCTTTCCGGTCTGATGAGTCCGTGAGGACGAAACAGCCTCTACAAATAATTTTGTTTAA
P3	RBS	CTTTACGAGGGCGATCCT
S2	RBS	GAGTCTATGGACTATGTTTTCACATATGAGATACCAGG
B2	RBS	CTATGGACTATGTTTTTCAAAGACGAAAAACTACTAG
E1	RBS	CCCCCCGAGGAGTAGCAC
F1	RBS	CTATGGACTATGTTTTCACATACGAGGGGGATTAG
H1	RBS	ACCCCCGAG
A1	RBS	AATGTTCCCTAATAATCAGCAAAGAGGTTACTAG
BBa_B0064	RBS	TACTAGAGAAAGAGGGGAAATACTAG
phlF	Gene	ATGGCACGTACCCCGAGCCGTAGCAGCATTGGTAGCCTGCGTAGTCCGCATACCCATAAAGCAATTCTGACCAGCACCATTGAAATCCTGAAAGAATGTGGTTATAGCGGTCTGAGCATTGAAAGCGTTGCACGTCGTGCCGGTGCAAGCAAACCGACCATTTATCGTTGGTGGACCAATAAAGCAGCACTGATTGCCGAAGTGTATGAAAATGAAAGCGAACAGGTGCGTAAATTTCCGGATCTGGGTAGCTTTAAAGCCGATCTGGATTTTCTGCTGCGTAATCTGTGGAAAGTTTGGCGTGAAACCATTTGTGGTGAAGCATTTCGTTGTGTTATTGCAGAAGCACAGCTGGACCCTGCAACCCTGACCCAGCTGAAAGATCAGTTTATGGAACGTCGTCGTGAGATGCCGAAAAAACTGGTTGAAAATGCCATTAGCAATGGTGAACTGCCGAAAGATACCAATCGTGAACTGCTGCTGGATATGATTTTTGGTTTTTGTTGGTATCGCCTGCTGACCGAACAGCTGACCGTTGAACAGGATATTGAAGAATTTACCTTCCTGCTGATTAATGGTGTTTGTCCGGGTACACAGCGTTAA
srpR	Gene	ATGGCACGTAAAACCGCAGCAGAAGCAGAAGAAACCCGTCAGCGTATTATTGATGCAGCACTGGAAGTTTTTGTTGCACAGGGTGTTAGTGATGCAACCCTGGATCAGATTGCACGTAAAGCCGGTGTTACCCGTGGTGCAGTTTATTGGCATTTTAATGGTAAACTGGAAGTTCTGCAGGCAGTTCTGGCAAGCCGTCAGCATCCGCTGGAACTGGATTTTACACCGGATCTGGGTATTGAACGTAGCTGGGAAGCAGTTGTTGTTGCAATGCTGGATGCAGTTCATAGTCCGCAGAGCAAACAGTTTAGCGAAATTCTGATTTATCAGGGTCTGGATGAAAGCGGTCTGATTCATAATCGTATGGTTCAGGCAAGCGATCGTTTTCTGCAGTATATTCATCAGGTTCTGCGTCATGCAGTTACCCAGGGTGAACTGCCGATTAATCTGGATCTGCAGACCAGCATTGGTGTTTTTAAAGGTCTGATTACCGGTCTGCTGTATGAAGGTCTGCGTAGCAAAGATCAGCAGGCACAGATTATCAAAGTTGCACTGGGTAGCTTTTGGGCACTGCTGCGTGAACCGCCTCGTTTTCTGCTGTGTGAAGAAGCACAGATTAAACAGGTGAAATCCTTCGAATAA
bm3R1	Gene	ATGGAAAGCACCCCGACCAAACAGAAAGCAATTTTTAGCGCAAGCCTGCTGCTGTTTGCAGAACGTGGTTTTGATGCAACCACCATGCCGATGATTGCAGAAAATGCAAAAGTTGGTGCAGGCACCATTTATCGCTATTTCAAAAACAAAGAAAGCCTGGTGAACGAACTGTTTCAGCAGCATGTTAATGAATTTCTGCAGTGTATTGAAAGCGGTCTGGCAAATGAACGTGATGGTTATCGTGATGGCTTCATCACATTTTTGAAGGTATGGTGACCTTTACCAAAAATCATCCGCGTGCACTGGGTTTTATCAAAACCCATAGCCAGGGCACCTTTCTGACCGAAGAAAGCCGTCTGGCATATCAGAAACTGGTTGAATTTGTGTGCACCTTTTTTCGTGAAGGTCAGAAACAGGGTGTGATTCGTAATCTGCCGGAAAATGCACTGATTGCAATTCTGTTTGGCAGCTTTATGGAAGTGTATGAAATGATCGAGAACGATTATCTGAGCCTGACCGATGAACTGCTGACCGGTGTTGAAGAAAGCCTGTGGGCAGCACTGAGCCGTCAGAGCTAA
betI	Gene	ATGCCGAAACTGGGTATGCAGAGCATTCGTCGTCGTCAGCTGATTGATGCAACCCTGGAAGCAATTAATGAAGTTGGTATGCATGATGCAACCATTGCACAGATTGCACGTCGTGCCGGTGTTAGCACCGGTATTATTAGCCATTATTTCCGCGATAAAAACGGTCTGCTGGAAGCAACCATGCGTGATATTACCAGCCAGCTGCGTGATGCAGTTCTGAATCGTCTGCATGCACTGCCGCAGGGTAGCGCAGAACAGCGTCTGCAGGCAATTGTTGGTGGTAATTTTGATGAAACCCAGGTTAGCAGCGCAGCAATGAAAGCATGGCTGGCATTTTGGGCAAGCAGCATGCATCAGCCGATGCTGTATCGTCTGCAGCAGGTTAGCAGTCGTCGTCTGCTGAGCAATCTGGTTAGCGAATTTCGTCGTGAACTGCCTCGTGAACAGGCACAAGAGGCAGGTTATGGTCTGGCAGCACTGATTGATGGTCTGTGGCTGCGTGCAGCACTGAGCGGTAAACCGCTGGATAAAACCCGTGCAAATAGCCTGACCCGTCATTTTATCACCCAGCATCTGCCGACCGATTAA
ameR	Gene	ATGAACAAAACCATTGATCAGGTGCGTAAAGGTGATCGTAAAAGCGATCTGCCGGTTCGTCGTCGTCCGCGTCGTAGTGCCGAAGAAACCCGTCGTGATATTCTGGCAAAAGCCGAAGAACTGTTTCGTGAACGTGGTTTTAATGCAGTTGCCATTGCAGATATTGCAAGCGCACTGAATATGAGTCCGGCAAATGTGTTTAAACATTTTAGCAGCAAAAACGCACTGGTTGATGCAATTGGTTTTGGTCAGATTGGTGTTTTTGAACGTCAGATTTGTCCGCTGGATAAAAGCCATGCACCGCTGGATCGTCTGCGTCATCTGGCACGTAATCTGATGGAACAGCATCATCAGGATCATTTCAAACACATACGGGTTTTTATTCAGATCCTGATGACCGCCAAACAGGATATGAAATGTGGCGATTATTACAAAAGCGTGATTGCAAAACTGCTGGCCGAAATTATTCGTGATGGTGTTGAAGCAGGTCTGTATATTGCAACCGATATTCCGGTTCTGGCAGAAACCGTTCTGCATGCACTGACCAGCGTTATTCATCCGGTTCTGATTGCACAAGAAGATATTGGTAATCTGGCAACCCGTTGTGATCAGCTGGTTGATCTGATTGATGCAGGTCTGCGTAATCCGCTGGCAAAATAA

hlyIIR	Gene	ATGAAATACATCCTGTTTGAGGTGTGCGAAATGGGTAAAAGCCGTGAACAGACCATGGAAAATATTCTGAAAG<br>CAGCCAAAAAGAAATTCGGCGAACGTGGTTATGAAGGCACCAGCATTCAAGAAATTACCAAAGAAGCCAAAGT<br>TAACGTTGCAATGGCCAGCTATTACTTTAATGGCAAAGAGAACCTGTACTACGAGGTGTTCAAAAAATACGGT<br>CTGGCAAATGAACTGCCGAACTTTCTGGAAAAAAACCAGTTTAATCCGATTAATGCCCTGCGTGAATATCTGA<br>CCGTTTTTACCACCCACATTAAAGAAAATCCGGAAATTGGCACCCTGGCCTATGAAGAAATTATCAAAGAAAG<br>CGCACGCCTGGAAAAAATCAAACCGTATTTTATCGGCAGCTTCGAACAGCTGAAAGAAATTCTGCAAGAGGGT<br>GAAAAACAGGGTGTGTTTCACTTTTTTAGCATCAACCATACCATCCATTGGATTACCAGCATTGTTCTGTTTC<br>CGAAATTCAAAAAATTCATCGATAGCCTGGGTCCGAATGAAACCAATGATACCAATCATGAATGGATGCCGGA<br>AGATCTGGTTAGCCGTATTATTAGCGCACTGACCGATAAACCGAACATTTAA
amtR	Gene	ATGGCAGGCGCAGTTGGTCGTCCGCGTCGTAGTGCACCGCGTCGTGCAGGTAAAAATCCGCGTGAAGAAATTC<br>TGGATGCAAGCGCAGAACTGTTTACCCGTCAGGGTTTTGCAACCACCAGTACCCATCAGATTGCAGATGCAGT<br>TGGTATTCGTCAGGCAAGCCTGTATTATCATTTTCCGAGCAAAACCGAAATCTTTCTGACCCTGCTGAAAAGC<br>ACCGTTGAACCGAGCACCGTTCTGGCAGAAGATCTGAGCACCCTGGATGCAGGTCCGGAAATGCGTCTGTGGG<br>CAATTGTTGCAAGCGAAGTTCGTCTGCTGCTGAGCACCAAATGGAATGTTGGTCGTCTGTATCAGCTGCCGAT<br>TGTTGGTAGCGAAGAATTTGCAGAATATCATAGCCAGCGTGAAGCACTGACCAATGTTTTTCGTGATCTGGCA<br>ACCGAAATTGTTGGTGATGATCCGCGTGCAGAACTGCCGTTTCATATTACCATGAGCGTTATTGAAATGCGTC<br>GCAATGATGGTAAAATTCCGAGTCCGCTGAGCGCAGATAGCCTGCCGGAAACCGCAATTATGCTGGCAGATGC<br>AAGCCTGGCAGTTCTGGGTGCACCGCTGCCTGCAGATCGTGTTGAAAAAACCCTGGAACTGATTAAACAGGCA<br>GATGCAAAATAA
Yfp	Gene	ATGGTGAGCAAGGGCGAGGAGCTGTTCACCGGGGTGGTGCCCATCCTGGTCGAGCTGGACGGCGACGTAAACG<br>GCCACAAGTTCAGCGTGTCCGGCGAGGGCGAGGGCGATGCCACCTACGGCAAGCTGACCCTGAAGTTCATCTG<br>CACCACAGGCAAGCTGCCCGTGCCCTGGCCCACCCTCGTGACCACCTTCGGCTACGGCCTGCAATGCTTCGCC<br>CGCTACCCCGACCACATGAAGCTGCACGACTTCTTCAAGTCCGCCATGCCCGAAGGCTACGTCCAGGAGCGCA<br>CCATCTTCTTCAAGGACGACGGCAACTACAAGACCCGCGCCGAGGTGAAGTTCGAGGGCGACACCCTGGTGAA<br>CCGCATCGAGCTGAAGGGCATCGACTTCAAGGAGGACGGCAACATCCTGGGGCACAAGCTGGAGTACAACTAC<br>AACAGCCACAACGTCTATATCATGGCCGACAAGCAGAAGAACGGCATCAAGGTGAACTTCAAGATCCGCCACA<br>ACATCGAGGACGGCAGCGTGCAGCTCGCCGACCACTACCAGCAGAACACCCCAATCGGCGACGGCCCCGTGCT<br>GCTGCCCGACAACCACTACCTTAGCTACCAGTCCGCCCTGAGCAAAGACCCCAACGAGAAGCGCGATCACATG<br>GTCCTGCTGGAGTTCGTGACCGCCGCCGGGATCACTCTCGGCATGGACGAGCTGTACAAGTAA
ECK120033737	Terminator	GGAAACACAGAAAAAAGCCCGCACCTGACAGTGCGGGCTTTTTTTTTCGACCAAAGG
ECK120029600	Terminator	TTGAGAAGAGAAAAGAAAACCGCCGATCCTGTCCACCGCATTACTGCAAGGTAGTGGACAAGACCGGCGGTCT<br>TAAGTTTTTTGGCTGAA
L3S2P11	Terminator	CTCGGTACCAAATTCCAGAAAAGAGACGCTTTCGAGCGTCTTTTTTCGTTTTGGTCC
L3S3P11	Terminator	CCAATTATTGAACACCCTTCGGGGTGTTTTTTTGTTTCTGGTCTACC
L3S3P31	Terminator	CCAATTATTGAACACCCTAACGGGTGTTTTTTTTTTTTTGGTCTACC
ECK120033736	Terminator	AACGCATGAGAAAGCCCCCGGAAGATCACCTTCCGGGGGCTTTTTTATTGCGC
L3S2P55	Terminator	CTCGGTACCAAAGACGAACAATAAGACGCTGAAAAGCGTCTTTTTTCGTTTTGGTCC
L3S2P21	Terminator	CTCGGTACCAAATTCCAGAAAAGAGGCCTCCCGAAAGGGGGGCCTTTTTTCGTTTTGGTCC

Supplementary References:

1 Nielsen, A. A. *et al.* Genetic circuit design automation. *Science* **352**, aac7341, doi:10.1126/science.aac7341 (2016).

2 Meyer, A. J., Segall-Shapiro, T. H., Glassey, E., Zhang, J. & Voigt, C. A. Escherichia coli "Marionette" strains with 12 highly optimized small-molecule sensors. *Nat Chem Biol* **15**, 196-204, doi:10.1038/s41589-018-0168-3 (2019).

3 Andrews, L. B., Nielsen, A. A. K. & Voigt, C. A. Cellular checkpoint control using programmable sequential logic. *Science* **361**, doi:10.1126/science.aap8987 (2018).

4 Gorochowski, T. E. *et al.* Genetic circuit characterization and debugging using RNA-seq. *Mol Syst Biol* **13**, 952, doi:10.15252/msb.20167461 (2017).

5 Crooks, G. E., Hon, G., Chandonia, J. M. & Brenner, S. E. WebLogo: a sequence logo generator. *Genome Res* **14**, 1188-1190, doi:10.1101/gr.849004 (2004).

6 Clifton, K. P. *et al.* The genetic insulator RiboJ increases expression of insulated genes. *J Biol Eng* **12**, 23, doi:10.1186/s13036-018-0115-6 (2018).

7 Lorenz, R. *et al.* ViennaRNA Package 2.0. *Algorithms Mol Biol* **6**, 26, doi:10.1186/1748-7188-6-26 (2011).

8 Stanton, B. C. *et al.* Genomic mining of prokaryotic repressors for orthogonal logic gates. *Nat Chem Biol* **10**, 99-105, doi:10.1038/nchembio.1411 (2014).

9 Needleman, S. B. & Wunsch, C. D. A general method applicable to the search for similarities in the amino acid sequence of two proteins. *J Mol Biol* **48**, 443-453, doi:10.1016/0022-2836(70)90057-4 (1970).
