# Supplementary material for: Genetic circuit characterization by inferring RNA polymerase movement and ribosome usage
Source: Nat Commun. 2020 Oct 5;11:5001. doi: 10.1038/s41467-020-18630-2 (PMC7536230; doi:10.1038/s41467-020-18630-2)
Supplement: Supplementary file 3 — Description of Additional Supplementary Files [file 41467_2020_18630_MOESM3_ESM.docx]

**Description of Additional Supplementary Files**

**Supplementary Data 1**

The measured FPKM, ribosome density (RD), and proteome fraction of all the proteins in the circuit and genome across 8 induction states of the circuit and the control cell.
