# Supplementary material for: Genetic circuit characterization by inferring RNA polymerase movement and ribosome usage
Source: Nat Commun. 2020 Oct 5;11:5001. doi: 10.1038/s41467-020-18630-2 (PMC7536230; doi:10.1038/s41467-020-18630-2)
Supplement: Supplementary file 5 — Reporting Summary [file 41467_2020_18630_MOESM5_ESM.pdf]

nature research

Corresponding author(s): Christopher A. Voigt

Last updated by author(s): Aug 27, 2020

# Reporting Summary

Nature Research wishes to improve the reproducibility of the work that we publish. This form provides structure for consistency and transparency in reporting. For further information on Nature Research policies, see Authors & Referees and the Editorial Policy Checklist .

## Statistics

For all statistical analyses, confirm that the following items are present in the figure legend, table legend, main text, or Methods section.

| n/a | Confirmed | |
|---|---|---|
| ☐ | ☒ | The exact sample size ($n$) for each experimental group/condition, given as a discrete number and unit of measurement |
| ☐ | ☒ | A statement on whether measurements were taken from distinct samples or whether the same sample was measured repeatedly |
| ☒ | ☐ | The statistical test(s) used AND whether they are one- or two-sided<br>*Only common tests should be described solely by name; describe more complex techniques in the Methods section.* |
| ☒ | ☐ | A description of all covariates tested |
| ☐ | ☒ | A description of any assumptions or corrections, such as tests of normality and adjustment for multiple comparisons |
| ☐ | ☒ | A full description of the statistical parameters including central tendency (e.g. means) or other basic estimates (e.g. regression coefficient) AND variation (e.g. standard deviation) or associated estimates of uncertainty (e.g. confidence intervals) |
| ☒ | ☐ | For null hypothesis testing, the test statistic (e.g. $F$, $t$, $r$) with confidence intervals, effect sizes, degrees of freedom and $P$ value noted<br>*Give P values as exact values whenever suitable.* |
| ☒ | ☐ | For Bayesian analysis, information on the choice of priors and Markov chain Monte Carlo settings |
| ☒ | ☐ | For hierarchical and complex designs, identification of the appropriate level for tests and full reporting of outcomes |
| ☒ | ☐ | Estimates of effect sizes (e.g. Cohen's $d$, Pearson's $r$), indicating how they were calculated |

*Our web collection on statistics for biologists contains articles on many of the points above.*

## Software and code

Policy information about availability of computer code

| | |
|---|---|
| Data collection | RBS Calculator software (version 2.0) was used to calculate the translation initiation rate (TIR) of RBSs. ViennaRNA software (version 1.8.5) was used to calculate the RNA folding energy and structure. Cello software (version 1.0) was used to predict the circuit promoter activities. WebLogo software (version 2.8.2) was used for motif identification. BD FACSDiva software (version 8.0.3) was used to acquire flow cytometry data. |
| Data analysis | FlowJo software (version 9) was used to analyze fluorescence data. Bowtie software (v1.1.2) was used for sequence alignment. EdgeR software in R (version 3.4.4) was used for differential gene expression analysis. All other parameterization of biological parts were performed using custom python scripts.<br><br>Python scripts that implement the complete characterization of genetic circuits, sensitivity analysis, and dynamic model of genetic circuits are released as open-source software under the MIT license (GitHub repository: https://github.com/VoigtLab/Comprehensive_Genetic_Circuit_Analysis). |

For manuscripts utilizing custom algorithms or software that are central to the research but not yet described in published literature, software must be made available to editors/reviewers. We strongly encourage code deposition in a community repository (e.g. GitHub). See the Nature Research guidelines for submitting code & software for further information.

## Data

Policy information about availability of data

All manuscripts must include a data availability statement. This statement should provide the following information, where applicable:

- Accession codes, unique identifiers, or web links for publicly available datasets
- A list of figures that have associated raw data
- A description of any restrictions on data availability

Data supporting the findings of this study are available within the paper and in Supplementary Data. RNA-seq and ribosome profiling data collected in this study

were deposited to Gene Expression Omnibus [https://www.ncbi.nlm.nih.gov/geo/query/acc.cgi?acc=GSE152664] under the accession number GSE152664. EcoCyc database (https://ecocyc.org/) was used to obtain a list of all unique DNA-binding proteins in E. coli MG1655 genome. DNA sequence of E. coli DH10B genome (NC_010473.1) was obtained from NCBI (https://www.ncbi.nlm.nih.gov/nuccore/NC_010473).

# Field-specific reporting

Please select the one below that is the best fit for your research. If you are not sure, read the appropriate sections before making your selection.

☒ Life sciences ☐ Behavioural & social sciences ☐ Ecological, evolutionary & environmental sciences

For a reference copy of the document with all sections, see nature.com/documents/nr-reporting-summary-flat.pdf

# Life sciences study design

All studies must disclose on these points even when the disclosure is negative.

| | |
|---|---|
| Sample size | No statistical methods were used to determine the sample sizes. However, 3 biological replicates were found to be sufficient for fluorescence measurements and cell doubling times since their coefficient of variations (std/mean) were less than 10%. |
| Data exclusions | No data was excluded from the analysis. However, mapped reads to the tRNAs regions were manually removed from all RNA-seq data to avoid potential biases in transcription profiles across the genome. |
| Replication | The genetic circuit was characterized under all 8 combinatorial induction states. 3 biological replicates were used for fluorescence measurements and cell doubling times of each state. One biological replicate was performed for RNA-seq and ribosome profiling of each state.<br><br>Circuit's output fluorescences and host cell's doubling times were highly reproducible across three replicates. All attempts at replication were successful.<br><br>Unfortunately, performing additional replicates for RNA-seq and ribosome profiling was not possible due to Covid-19 pandemic. However, although this is not the best measure for reproducibility, the performances of the circuit's parts and gates were found to be reproducible across different induction states of the circuit. |
| Randomization | Grouping was not relevant for this study, since each induction state of the circuit was analyzed individually and compared with other states. |
| Blinding | Blinding was not relevant for this study, since each induction state of the circuit was analyzed individually and compared with other states. |

# Reporting for specific materials, systems and methods

We require information from authors about some types of materials, experimental systems and methods used in many studies. Here, indicate whether each material, system or method listed is relevant to your study. If you are not sure if a list item applies to your research, read the appropriate section before selecting a response.

## Materials & experimental systems

| n/a | Involved in the study | |
|---|---|---|
| ☒ | ☐ | Antibodies |
| ☒ | ☐ | Eukaryotic cell lines |
| ☒ | ☐ | Palaeontology |
| ☒ | ☐ | Animals and other organisms |
| ☒ | ☐ | Human research participants |
| ☒ | ☐ | Clinical data |

## Methods

| n/a | Involved in the study | |
|---|---|---|
| ☒ | ☐ | ChIP-seq |
| ☐ | ☒ | Flow cytometry |
| ☒ | ☐ | MRI-based neuroimaging |

## Flow Cytometry

### Plots

Confirm that:

☒ The axis labels state the marker and fluorochrome used (e.g. CD4-FITC).

☒ The axis scales are clearly visible. Include numbers along axes only for bottom left plot of group (a 'group' is an analysis of identical markers).

☒ All plots are contour plots with outliers or pseudocolor plots.

☒ A numerical value for number of cells or percentage (with statistics) is provided.

## Methodology

| | |
|---|---|
| Sample preparation | Circuit containing E. coli cells were brought to exponential phase of growth, followed by 5 hours induction, after which 20 µl of culture was added to 180 µl 1x PBS solution with 2 mg/ml kanamycin to stop translation and cell growth, and the mixture was incubated for one hour before fluorescence was measured using flow cytometry. |
| Instrument | Fluorescence was measured using an LSRII Fortessa flow cytometer (BD Biosciences, San Jose, CA) using BD FACSDiva software. |
| Software | The flow cytometer software FlowJo (TreeStar, Inc., Ashland, OR) was used to calculate the median YFP fluorescence values. |
| Cell population abundance | More than 10,000 gated events were collected for each sample. |
| Gating strategy | Events with YFP fluorescence > 0, 350 < FSC-A < 10000, and 3500 < SSC-A < 100000 were gated. |

☒ Tick this box to confirm that a figure exemplifying the gating strategy is provided in the Supplementary Information.
